# Identifying behaviour change techniques in 287 randomized controlled trials of audit and feedback interventions targeting practice change among healthcare professionals

**DOI:** 10.1186/s13012-023-01318-8

**Published:** 2023-11-21

**Authors:** Jacob Crawshaw, Carly Meyer, Vivi Antonopoulou, Jesmin Antony, Jeremy M. Grimshaw, Noah Ivers, Kristin Konnyu, Meagan Lacroix, Justin Presseau, Michelle Simeoni, Sharlini Yogasingam, Fabiana Lorencatto

**Affiliations:** 1 Centre for Evidence-Based Implementation, Hamilton Health Sciences, Hamilton, ON Canada; 2 Department of Medicine, McMaster University, Hamilton, ON Canada; 3 Department of Clinical, Educational and Health Psychology, Centre for Behaviour Change, University College London, London, WC1E 7HB UK; 4 NIHR Policy Research Unit in Behavioural Science, Newcastle University, Baddiley-Clark Building, Richardson Road, Newcastle upon Tyne, NE2 4AX UK; 5 Women’s College Research Institute, Women’s College Hospital, Toronto, ON Canada; 6 Centre for Implementation Research, Ottawa Hospital Research Institute, Ottawa, Canada; 7 School of Epidemiology and Public Health, University of Ottawa, Ottawa, Canada; 8 Department of Health Services, Policy and Practice, Center for Evidence Synthesis in Health, Brown University School of Public Health, Brown University, Providence, Rhode Island, USA

**Keywords:** Audit and feedback, Quality improvement, Healthcare professionals, Practice change, Behaviour change techniques, Implementation

## Abstract

**Background:**

Audit and feedback (A&F) is among the most widely used implementation strategies, providing healthcare professionals with summaries of their practice performance to prompt behaviour change and optimize care. Wide variability in effectiveness of A&F has spurred efforts to explore why some A&F interventions are more effective than others. Unpacking the variability of the content of A&F interventions in terms of their component behaviours change techniques (BCTs) may help advance our understanding of how A&F works best. This study aimed to systematically specify BCTs in A&F interventions targeting healthcare professional practice change.

**Methods:**

We conducted a directed content analysis of intervention descriptions in 287 randomized trials included in an ongoing Cochrane systematic review update of A&F interventions (searched up to June 2020). Three trained researchers identified and categorized BCTs in all trial arms (treatment & control/comparator) using the 93-item BCT Taxonomy version 1. The original BCT definitions and examples in the taxonomy were adapted to include A&F-specific decision rules and examples. Two additional BCTs (‘Education (unspecified)’ and ‘Feedback (unspecified)’) were added, such that 95 BCTs were considered for coding.

**Results:**

In total, 47/95 BCTs (49%) were identified across 360 treatment arms at least once (median = 5.0, *IQR* = 2.3, range = 1-29). The most common BCTs were ‘Feedback on behaviour’ (present 89% of the time; e.g. feedback on drug prescribing), ‘Instruction on how to perform the behaviour’ (71%; e.g. issuing a clinical guideline), ‘Social comparison’ (52%; e.g. feedback on performance of peers), ‘Credible source’ (41%; e.g. endorsements from respected professional body), and ‘Education (unspecified)’ (31%; e.g. giving a lecture to staff). A total of 130/287 (45%) control/comparator arms contained at least one BCT (median = 2.0, *IQR* = 3.0, range = 0–15 per arm), of which the most common were identical to those identified in treatment arms.

**Conclusions:**

A&F interventions to improve healthcare professional practice include a moderate range of BCTs, focusing predominantly on providing behavioural feedback, sharing guidelines, peer comparison data, education, and leveraging credible sources. We encourage the use of our A&F-specific list of BCTs to improve knowledge of what is being delivered in A&F interventions. Our study provides a basis for exploring which BCTs are associated with intervention effectiveness.

**Trial registrations:**

N/A.

**Supplementary Information:**

The online version contains supplementary material available at 10.1186/s13012-023-01318-8.

Contributions to the literature This is the most comprehensive synthesis of the behaviour change content in randomized controlled trials of audit and feedback interventions and implementation interventions more generally that target practice change among healthcare professionals to date.

Our adapted taxonomy includes behaviour change techniques operationalized for the audit and feedback context, which will help both the reporting and design of audit and feedback interventions moving forward.

Now that the behaviour change techniques present in audit and feedback interventions have been specified, along with those which may be underutilized; it is possible to begin exploring which techniques and/or combinations of techniques are associated with intervention effectiveness. This will inform the optimization of the design and delivery of audit and feedback for practice change.

## Background

Audit and feedback (A&F) is defined as any summary of clinical performance of healthcare over a specified time period [1, 2]. A&F is one of the most widely used implementation strategies to improve quality and delivery of healthcare, either on its own or as part of a multicomponent intervention [3]. Indeed, there have been hundreds of randomized controlled trials (RCTs) evaluating the effect of A&F on healthcare professional practice change [1, 4]. A Cochrane systematic review of these trials identified that A&F typically achieves modest, yet worthwhile, improvements in compliance with desired clinical practice across a range of clinical areas. However, effectiveness varies substantially across A&F interventions (median 4.3% improvement, interquartile range 0.5 to 16%) [1]. A&F interventions have been designed and delivered in many different ways, which may in part contribute to observed variability in outcomes [5]. Attempts to unpack how variation in design and delivery of A&F might contribute to heterogeneity have identified that A&F is more effective when baseline performance is low, feedback is provided more than once, is delivered by a colleague or supervisor, and in both verbal and written formats [1]. However, these characteristics pertain mainly to context (e.g. low baseline performance) and mode of intervention delivery (i.e. how, and how much), rather than the content of the intervention (i.e. what). Comparatively, less is known about the components constituting the content of A&F interventions and how differences in the content may underpin variation in intervention effectiveness.

The application of behavioural science frameworks can help support the specification of the content of A&F interventions targeting healthcare professional practice. To do this, one must begin with conceptualizing healthcare professional practice as a form of human behaviour [6, 7]. Healthcare professional practices commonly targeted for A&F such as appropriate prescribing, radiology or laboratory test utilization, and management of patients with chronic conditions are all centred around behaviour [1]. In turn, A&F can be conceptualized as a form of behaviour change intervention, which has been hypothesized to work by healthcare professionals being prompted to modify their practice (i.e. change their behaviour) when given performance feedback showing their clinical practice is inconsistent with a desirable target (e.g. a guideline, best practice) [1].

Behaviour change interventions are typically complex and often contain multiple, interacting component behaviour change techniques (BCTs) [8]. BCTs are defined as ‘observable, replicable and irreducible components of an intervention that are designed to alter or redirect causal processes regulating behaviour’ [9]. Over the past decade, there has been a concerted effort to develop taxonomies of BCTs, the most recent and comprehensive of which is the BCT Taxonomy version 1 (BCTTv1). BCTTv1 includes 93 unique BCTs, each with a label, definition, and example of how that BCT could be operationalized and delivered [9]. These BCTs are organized into 16 clusters representing the potential mechanisms through which the BCTs may serve to change behaviour (e.g. shaping knowledge, associations, feedback and monitoring).

The BCTTv1 can be applied as a framework for identifying and classifying component BCTs in behaviour change interventions using standardized terminology to identify the ‘active ingredients’ of interventions. Specifying component BCTs using the BCTTv1 facilitates evidence synthesis and comparison across trials of behaviour change interventions and also supports replication and scalability [8]. It also provides a basis for examining the association between the presence of component BCTs and their associations with intervention outcomes, using techniques such as meta-regressions [10]. Indeed, an increasing number of systematic reviews have been conducted using the BCTTv1 as a framework for specifying BCTs in interventions targeting a wide range of behaviours, from physical activity [11] to smoking cessation [12] to attendance for diabetic retinopathy screening [13]. However, most BCT-informed reviews to date have focused on patient and general population behaviours, and comparatively, fewer reviews have been conducted to identify the active ingredients of implementation interventions targeting healthcare professional behaviour change. One example of the latter is a review that applied BCTTv1 to specify the components of 23 trials of implementation interventions to improve care and management of diabetes [14]. Commonly used BCTs targeting healthcare professional behaviour included but were not limited to the following: ‘Adding objects to the environment’ (e.g. introducing colourful folders with foot decals to identify intervention patients), ‘Prompts/cues’ (e.g. monthly telephone calls and newsletters to keep pharmacists engaged and motivated), ‘Instruction on how to perform the behaviour’ (e.g. educational booklet, lectures, workshops on diabetes care), ‘Credible source’ (e.g. linking recommended messages to supporting peer-reviewed publications/evidence and local and national guidelines), and ‘Goal setting (outcome)’ (e.g. setting targets such as % of patients with HbA1c levels below a certain threshold) [14].

We also sought to examine patterns in the number of BCTs included in A&F interventions over time (both the average number of BCTs across studies per publication year and the maximum number reported per publication year). Recent years have seen increased emphasis on better and more comprehensive reporting of complex interventions, exemplified by checklists such as CONSORT and the TIDIeR [15, 16]. There has also been increased advocacy for the use of behaviour change theories and frameworks when designing interventions to change healthcare professional practice [17]. As such, we hypothesized that more recently conducted studies would be more likely to incorporate and report a greater number of BCTs in their interventions compared to older studies, given advances in the A&F trial literature, and calls for better reporting of behavioural trials.

One challenge to potentially applying the BCTTv1 to code implementation interventions targeting healthcare professional behaviours is that many of the examples included in the current taxonomy focus on patient and general population behaviours (i.e. diet, physical activity, smoking, alcohol consumption, medication adherence). This in turn can make it challenging to conceptualize how each BCT could be delivered in the different context of behaviour change among healthcare professionals. Therefore, as part of this study, we sought to adapt the BCTTv1 to include modified examples of how BCTs could be operationalized in the healthcare professional behaviour change context and A&F specifically. We anticipate this could be useful not only for future coding studies/reviews but also to inform the design and delivery of A&F interventions going forward by providing examples of how BCTs could be delivered as part of an A&F intervention. We believe there is an opportunity to leverage such frameworks and methods to improve our knowledge about exactly what content is being delivered in A&F interventions (albeit limited by the level of reporting among studies) to ultimately help optimize, reproduce, scale, and spread effective versions of A&F for healthcare professionals and provide a basis for subsequent investigations of which component BCTs contribute to more effective A&F.

### Aims

The aims of this study were therefore as follows:

- Adapt the BCTTv1 definitions and examples to facilitate the identification of BCTs within A&F interventions directed at changing the behaviour of healthcare professionals.
- Apply the BCTTv1 to identify and specify the behaviour change content of A&F interventions targeting healthcare professional practice change and assess patterns in the number of BCTs included in A&F interventions over time.

## Methods

### Present study design

Our study was conducted alongside an ongoing update of the 2012 Cochrane systematic review investigating the effectiveness of A&F interventions on healthcare professional practice [1, 18]. We performed a directed content analysis of intervention descriptions from 287 RCTs (including cluster RCTs) included in the update of the review which searched the literature up to June 2020 (see Appendix 1 for list of included studies). We examined the behaviour change content of all trial arms (i.e. treatment & control/comparator).

### Overview of trials of A&F included in our study

Details of the search strategy and eligibility criteria for the included A&F trials are reported elsewhere [1, 18]. Studies were RCTs or cluster RCTs of healthcare professional interventions which included A&F to target practice change (either standalone A&F and/or A&F plus co-interventions), which will be referred to as ‘A&F interventions’ hereafter. Table 1 provides an overview of key study characteristics from trials included in our study. In summary, most trials were conducted in the USA (40%), targeted physicians (92%), adopted a two-arm trial design (77%), and were delivered in primary care settings (56%). The most targeted healthcare professional behaviours were prescribing (51%), testing/exams (32%), treatment decision/action (19%), screening (17%), and counselling (17%).

**Table 1:** Summary of the key study characteristics of 287 trials of A&F interventions targeting practice change among healthcare professionals

Study characteristics	Number of trials (%)
**Publication year**	
2016–2020	75 (26%)
2011–2015	52 (18%)
2006–2010	46 (16%)
2001–2005	53 (19%)
1996–2000	29 (10%)
Before 1996	32 (11%)
**Country**	
USA	116 (40%)
UK or Ireland	35 (12%)
Canada	29 (10%)
Australia or New Zealand	18 (6%)
Other	89 (31%)
**Number of trial arms**	
Two	222 (77%)
Three	37 (13%)
Four	25 (9%)
More than four	3 (1%)
**Clinical setting**	
Primary care	161 (56%)
Hospital inpatient	65 (23%)
Other outpatient clinic	24 (8%)
Community care	17 (6%)
Other/mixed	20 (7%)
**Medical specialty**	
General practitioner/family physician	167 (58%)
Internists	59 (21%)
Other	61 (21%)
**Targeted healthcare professionals (could include more than one)**	
Physician	263 (92%)
Nurses	51 (18%)
Pharmacists	8 (3%)
Other	43 (15%)
**Targeted healthcare professional behaviour (could include more than one)**	
Prescribing	139 (51%)
Testing/exams	88 (32%)
Treatment decision/action	53 (19%)
Screening	49 (18%)
Counselling	47 (17%)
Immunization	26 (9%)
Referrals	16 (6%)
Diagnosis	11 (4%)
Other	6 (2%)

### Behaviour change technique coding

#### Coding framework

The original published BCTTv1 was used as an initial coding framework for our directed content analysis. Generic coding instructions, definitions, and examples from the BCTTv1 were adapted to include examples relevant to healthcare professional behaviour change and specifically within the A&F context. This was done iteratively throughout the coding process, whereby we generated A&F-specific heuristics, extracted examples of BCTs identified within our A&F trial dataset, and added these as examples to the original taxonomy. We developed coding heuristics for 47 BCTs covering what to code and what not to code, based on regular discussions between our coding team and the wider research team where necessary (see the ‘Coding procedure’ section for further details). Our coding processes were informed by the methods used to develop BCT coding frameworks reported in previous review studies of healthcare professional behaviour change interventions [14, 19]. The final coding framework is available in Appendix 2.

### Coding procedure

We used NVivo (version 12; QSR International Pty Ltd., Doncaster, Australia, 2018) to conduct BCT coding. For each trial, we coded intervention content for all study arms based on all relevant published source material (i.e. manuscripts, supplementary materials, and study protocols). As part of this process, intervention descriptions were read line by line, and BCTs from the BCTTv1 were rated as ‘present’ or ‘absent’. Given that A&F is often used in conjunction with additional components or as a co-intervention, all intervention content was coded (i.e. not just A&F). For studies that evaluated combinations of healthcare professional- and patient-targeted interventions, we only coded the healthcare professional-targeted interventions.

Within our A&F trial dataset, designating control/comparator arms could be a challenge given the variety of trial designs used, often including active control groups and head-to-head comparisons of A&F interventions (e.g. A&F intervention vs. no intervention control, A&F intervention vs. co-intervention vs no intervention control, A&F intervention vs. A&F intervention, A&F intervention & co-intervention vs. A&F intervention only). As such, some control/comparator arms included components of A&F. We assigned study arms to treatment or control/comparator based primarily on how relative intervention content was reported in each paper. For studies without explicit control arms, we assigned the lowest intensity treatment arm as a control/comparator arm (e.g. education vs. education & academic detailing vs. education & academic detailing & A&F). Only one arm per study was assigned as a control/comparator. The coding team was comprised of three researchers (J. C., C. M., V. A.) with behavioural science expertise including coding the behavioural content of interventions and experience in healthcare professional behaviour change. To establish consistency in coding, the first 48 trials were triple coded, the next 101 trials were double coded, and then the final 141 trials were single coded with 20% double coded as a spot-checking process. Coding was mostly done in blocks of 10 to 15 papers at a time. Any discrepancies were discussed during regular consensus meetings of the coding team; when consensus could not be reached, the wider study team (with expertise and experience in both A&F research, behaviour change, and prior experience with working with BCTTv1) were consulted. The coding framework was refined accordingly as needed following consensus and wider team discussions.

### Data analysis

We used descriptive statistics to summarize the number and type of BCTs identified in A&F interventions (median and interquartile range (IQR), mean and standard deviation (SD)). We compared the number and type of BCTs identified in trial treatment arms versus control/comparator arms. To explore whether BCT frequencies changed over time, we also calculated the mean number of BCTs by study publication year. Studies were collapsed per year of publication and subsequently, mean BCTs for the treatment and control/comparator groups were calculated.

## Results

### Development of a BCT coding framework for A&F interventions

The resulting coding framework, which includes examples and adapted interventions relevant to healthcare professional behaviour change and A&F specifically, is available in Appendix 2. Table 2 provides an example of how an original BCT definition and example from the taxonomy was adapted during the coding framework development to relate to healthcare professional behaviour change and A&F.

**Table 2:** Example of adapting a behaviour change technique’s original definition and example to A&F and healthcare professional behaviour change context

Behaviour change technique	Original definition^a^	Original example^a^	A&F specific heuristics^b^	Examples related to A&F and healthcare professional behaviour change
**1.1 Goal setting (behaviour)**	Set or agree on a goal defined in terms of the behaviour to be achieved	Agree on a daily walking goal (e.g. 3 miles) with the person and reach agreement about the goal	Goal setting can be implied when clear behavioural targets, frequently based on clinical practice guidelines, are established and communicated with healthcare professionals prior to the receipt of feedback. Common examples of behavioural goals in the healthcare context relate to healthcare professionals referral and/or testing practices	‘Radiologists were able to insert their goals for changes they would like to make in their clinical practice, especially regarding recall rates, into a text field at the end of each module’ (Carney, 2012 [20])
	***Note:**** Only code Goal setting if there is sufficient evidence that goal set as part of intervention; if goal unspecified or a behavioural outcome, code 1.3, Goal setting (outcome); if the goal defines a specific context, frequency, duration, or intensity for the behaviour, also code 1.4, Action planning*			
		Set the goal of eating 5 pieces of fruit per day as specified in public health guidelines		
				‘Finally participants set their own, internal targets guided by the information presented to increase target’ (Roos-Blom, 2019 [21])
			Use content of reported outcomes to decide whether to code Goal setting (outcome) and/or Goal setting (behaviour). If unclear, code both (see 1.3)	
			*Key words*: Targets, metrics, standards, thresholds	

^a^From Michie S. et al. ‘The behavior change technique taxonomy (v1) of 93 hierarchically clustered techniques: building an international consensus for the reporting of behavior change interventions’. *Annals of behavioral medicine* 46.1 (2013): 81–95 [9]. ^b^Presseau, J. et al. ‘Using a behaviour change techniques taxonomy to identify active ingredients within trials of implementation interventions for diabetes care’. *Implementation Science* 10.1 (2015): 1–10 [14]

During the coding process, two key coding rules were established which helped shape our BCT coding framework. Firstly, A&F interventions are often evaluated in routine practice/real-world settings which typically requires concurrent implementation processes designed to support evaluation (e.g. supporting uptake and engagement, targeting intervention fidelity). Such implementation processes may include behaviour change content which could be captured at the BCT level (e.g. telephone calls from the research team to local coordinating team at a participating facility to troubleshoot around any issues with implementing the intervention during the trial or as a reminder to collect data could be coded as the BCTs ‘Social support (practical)’, ‘Problem-solving’, and/or ‘Prompts and cues’). Where possible, we established the coding rule that we did not code BCTs that were explicitly part of an implementation process (e.g. supporting evaluation, uptake, engagement, fidelity).

Secondly, we expected that feedback-related BCTs and education-related BCTs would feature prominently. Due to the typically limited detail and reporting of behaviour change intervention content [22], it was at times not possible to distinguish between the type of feedback provided and to in turn select between the BCTs ‘Feedback on behaviour’ (e.g. feedback on antibiotic prescribing) or ‘Feedback on outcome(s) of behaviour’ (e.g. feedback on infection rates). For example, some studies simply reported that they conducted an ‘A&F intervention’ with additional details absent. Therefore, we included a new BCT in the coding framework, ‘Feedback (unspecified)’ to account for this lack of specificity in study reports. Similarly, there were instances where intervention descriptions mentioned providing education or training to healthcare professionals but did not specify the content further (e.g. not clear whether the BCT ‘Instruction on how to perform the behaviour’ was provided or not). Frequently, authors referred to the provision of educational materials, learning modules, evidence summaries, and academic detailing, without any additional information. Therefore, we added another BCT named ‘Education (unspecified)’ to not miss this relevant intervention content. As such, a total of 95 BCTs were considered for coding (93 from the published BCTTv1 & two additional BCTs).

### Frequency of identified behaviour change techniques

A total of 287 studies were included, comprising of 360 treatment arms and 287 control/comparator arms.

### *Treatment arms (N* = *360)*

**Fig. 1:**
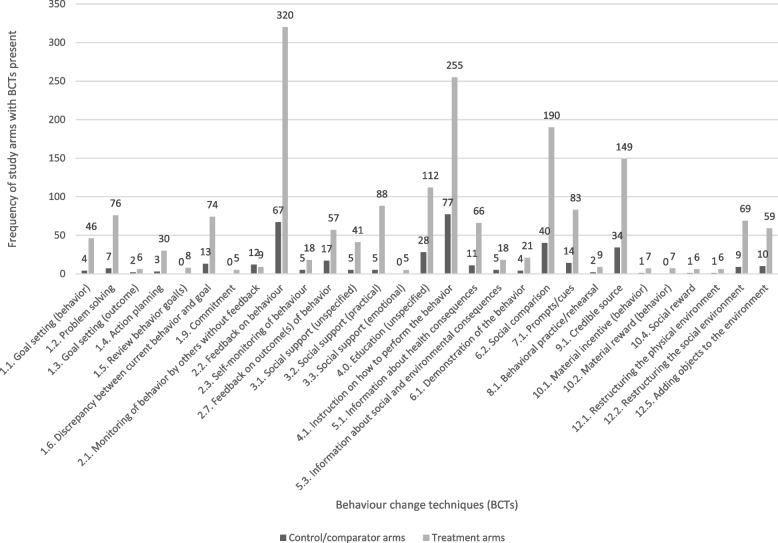
Frequency of the most coded BCTs (≥ 5 instances within treatment arms) in all study arms. Notes: Numbers above each bar represents BCT frequency

Table 3 lists the BCTs identified within treatment arms, their frequencies, and an example of how that BCT was delivered in one of the included A&F trials. The most frequently coded BCTs (≥ 5 instances within treatment arms) are depicted in Fig. 1. In summary, 47 out of a possible 95 BCTs (49%) were identified in the treatment arm of at least one A&F trial. BCTs were identified in 14/16 possible clusters of BCTs, although no BCTs were identified for the following taxonomy clusters: ‘regulation’ and ‘covert learning’.

**Table 3:** BCTs identified within 287 trials of A&F interventions targeting practice change among healthcare professionals

Taxonomy clusters	BCTs	Frequency in treatment arms (*N* = 360)	Example from treatment arms	Frequency in control/comparator arms (*N* = 287)	Example from control/comparator arms
*1. Goals and planning*	1.1 ‘Goal setting (behaviour)’	46	Avery et al. (2012): ‘Encourage the team to agree on an action plan with clear objectives’ [23]	4	Ornstein et al. (2004): ‘The medical director was encouraged to share the reports with others in the practice in order to stimulate motivation for improvement. The 90th percentile was selected as the performance target because it reflected a bold but achievable goal (at least 2 practices were at this level of performance at baseline)’ [24]
			Lemelin et al. (2001): ‘All practices were involved in meetings with the PF to identify opportunities for improvement, assess needs, and select priority areas and strategies for improving preventive care performance’ [25]		
			Ganz et al. (2005): ‘Assistance with an organizational strategy/goal meeting to review audit findings and select strategies’ [26]		
			Levi et al. (2020): ‘…sites setting own goals and interim targets for increasing tPA rate’ [27]		
			Harris et al. (2015): ‘…each practice reviewed their performance and set and reviewed goals specific to their individual circumstances and resources’ [28]		
	1.2 ‘Problem-solving’	76	Curtis et al. (2011): ‘Meetings with local champions allowed us to discuss barriers to quality end-of-life care in their units and strategize about ways to address those barriers’ [29]	7	Kaminski et al. (2016): ‘Discussion on barriers and solutions to improve ADR (adenoma detection rate)’ [30]
	1.3 ‘Goal setting (outcome)’	6	Ivers et al. (2013): ‘The worksheet was designed to facilitate goal-setting’ [31]	2	Goderis et al. (2010): ‘Targets were set at 7% for HbA1c, 130 mmHg for SBP and 100 mg/dl for LDL-C’ [32]
			Roos-Blom et al. (2019): ‘Finally participants set their own, internal targets guided by the information presented to increase target’ [21]		
	1.4 ‘Action planning’	30	Foy et al. (2004): ‘Immediately after, a brief meeting took place with lead consultants and other key individuals to formulate a local action plan’ [33]	3	Verstappen et al. (2003): ‘The next step was to try to implement the guidelines in their own practice, and at the end of each session, plans were drawn up for change, both at individual and group level. Subsequent meetings were used to evaluate whether targets had been met’ [34]
			Kennedy et al. (2015): ‘An action plan worksheet was completed/updated at each educational meeting, which outlined specific tasks and steps for implementing process/ policy changes’ [35]		
			Hogg et al. (2008): ‘Based on the practice’s care goals and their choice of tools, a plan or strategy was agreed upon with the facilitator for reaching the proposed goals’ [36]		
	1.5 ‘Review behaviour goal(s)’	8	Gude et al. (2016): ‘Next, the team discussed and reflected upon their most recent feedback report and created or updated their QI plan’ [37]	0	N/A
			Palmer et al. (1985): ‘At a staff meeting 3 months after evaluation findings had been discussed, department chiefs were asked to review the report of actions planned to improve care and to check progress in implementing these plans’ [38]		
	1.6 ‘Discrepancy between current behaviour and goal’	74	Wiggers et al. (2017): ‘Reports included comparison against target benchmarks’. [39]	13	Mold et al. (2008): ‘Benchmark rates (90th percentiles) were determined from clinician audits done as part of another study done in the previous year that involved 50 network clinicians’ [40]
	1.9 ‘Commitment’	5	Gascón Cánovas et al. (2009): ‘…committed themselves to perform a complete cycle of assessment and improvement’ [41]	0	N/A
			Shen et al. (2018): ‘The public commitment asked each of the participating village doctors in the intervention group to sign a letter of commitment and made the signed letter public by posting it on the walls of his or her clinic and printing it on the back of the patient takeaway information leaflet’ [42]		
*2. Feedback and monitoring*	2.0 ‘Feedback (unspecified)’ ^a^	4	Yano et al. (2008): ‘Each practice also received quarterly audit-and-feedback progress reports’ [43]	1	Yano et al. (2008): ‘…received audit-and-feedback reports’ [43]
			Bahrami et al. (2004): ‘Group 2 participated in Audit and Feedback (A and F)… The exact nature of the A and F was decided within each audit group’ [44]		
			Scales et al. (2016): ‘Audit-feedback using monthly enrolment reports of site-specific data compared with anonymous data from other hospitals’ [45]		
	2.1 ‘Monitoring of behaviour by others without feedback’	9	Wang et al. (2018): ‘The intervention cluster’s investigators or quality coordinator have access to view the level of implementation of predefined performance measures at any time (recommended once per week) and compare with previous performance and with performance by other clusters (not identified by name)’ [46]	12	Boet et al. (2018): ‘The control group had their performance audited, but no feedback was provided as per current hospital practice’ [47]
	2.2 ‘Feedback on behaviour’	320	Katz et al. (2004): ‘… group and confidential individual feedback on whether intake clinicians had assessed smoking status and whether they had provided cessation counseling’ [48]	67	Siriwardena et al. (2002): ‘Control practices undertook baseline data collection and received written feedback on their vaccination rates compared with other participating practices’ [49]
	2.3 ‘Self-monitoring of behaviour’	18	Clyne et al. (2015): ‘GPs were asked to conduct 1 review per patient using the web-based platform to guide them through the process’ [50]	5	Bonevski et al. (1999): ‘…to complete the practitioner checklist’ [51]
			Buffington et al. (1991): ‘Physicians and their staffs were asked to record all influenza immunizations given to patients aged 65 years or older, to tabulate on a weekly basis the cumulative total, and to calculate the percentage of the target population immunized’ [52]		
			Nace et al. (2020): ‘an active monitoring sheet designed to improve identification and documentation of signs and symptoms associated with the diagnosis of UTIs’ [53]		
	2.4 ‘Self-monitoring of outcome(s) of behaviour’	1	von Lengerke et al. (2019): ‘2.4 Self-monitoring of outcome(s) of behaviour, e.g. presentation of compliance rates and discussion of options for monitoring on wards’ [54]	1	von Lengerke et al. (2019): ‘2.4 Self-monitoring of outcome(s) of behaviour, e.g. presentation of compliance rates and discussion of options for monitoring on wards’ [54]
	2.5 ‘Monitoring of outcome(s) of behaviour without feedback’	0	N/A	2	Crotty et al. (2004): ‘Medication charts were reviewed for prescription and administration of any psychotropic and/or antihypertensive medication and use of aspirin or warfarin. Information was also collected on the number of falls and injurious falls in the last 12 months from incident report forms at each facility’ [55]
	2.7 ‘Feedback on outcome(s) of behaviour’	57	Boet et al. (2018): ‘The benchmarked feedback group had their performance audited and monthly benchmarked feedback was provided by email. Feedback included their individual performance outcomes’ [47]	17	Harris et al. (2013): ‘…summary chart audit data were presented and individual practice-specific “report cards” distributed’ [56]
			Hermans et al. (2013): ‘The benchmarking procedure comprised feedback given to each investigator regarding the level of control of the preset targets of their patients’ [57]		von Lengerke et al. (2019): ‘2.7 Feedback on outcome(s) of behaviour, e.g. feedback on hospital-wide NI rates’ [54]
*3. Social support*	3.1 ‘Social support (unspecified)’	41	Bregnhøj et al. (2009): ‘Afterwards, the GPs were contacted by telephone by a senior clinical pharmacologist (JS) to discuss any uncertainties concerning the recommendations given…’ [58]	5	von Lengerke et al. (2019): ‘3.1 Social support (unspecified), e.g. identification and forwarding of employees’ ideas for improvement’ [54]
			Campbell et al. (2006): ‘Facilitators attempted to maintain phone contact with clinics at least once a month for as long as these contacts seemed useful’ [59]		
	3.2 ‘Social support (practical)’	88	Harris et al. (2015): ‘Trained practice facilitators visited and met with practice staff (for at least three 1–2 h) to develop and support the implementation of a plan to improve the prevention of vascular disease in the practice population’ [28]	11	Ayieko et al. (2019): ‘…peer to peer networking through twice yearly meetings and a simple WhatsApp group’ [60]
	3.3 ‘Social support (emotional)’	5	von Lengerke et al. (2019): ‘3.3 Social support (emotional), e.g. active listening in feedback discussions to evoke reflection on balancing benefits and costs’ [54]	0	N/A
*4. Shaping knowledge*	4.0 ‘Education (unspecified)’ ^a^	112	Mertens et al. (2015): ‘At 6 months, clinics received a 30-min “booster” training’ [61]	28	Beeckman et al. (2013): ‘The standard protocol was presented by the senior nurse in a standardized 30 min group lecture (attended by all members of nursing staff)’ [62]
			Zwar et al. (1999): ‘An educational visit was undertaken with those trainees who at survey 2 were prescribing an antibiotic on more than one occasion for every ten URTI problems managed’ [63]		
					Ferguson et al. (2003): ‘Sites were informed that they may periodically receive supplemental educational reports in addition to the standard site-specific semi annual reports’ [64] Scholes et al. (2006): ‘This was accompanied by notification of a new guideline on the intranet guideline homepage… The posting included a summary of supporting evidence’ [65]
			Gude et al. (2016): ‘Educational outreach visits were conducted by one and typically lasted 2.5 h’ [37]		
			Kaboré et al. (2019): ‘Quarterly educational outreach visits’ [66]		
	4.1 ‘Instruction on how to perform the behaviour’	255	Awad et al. (2006): ‘They also received written specific recommendations for improvement according to their baseline prescribing quality levels’ [67]	77	Ayieko et al. (2019): ‘Clinicians in all hospitals were also supplied with updated protocol booklets that contained information on the new pneumonia guidance including specific pneumonia algorithms articulating the key clinical signs and how these are to be used in classification together with dosage tables for oral amoxicillin’ [60]
					DeVore et al. (2015): ‘For this study, the control hospitals continued to receive access to the usual on-demand reports, GWTG-HF quality improvement tools, and publicly available GWTG-HF webinars’ [68]
	4.2 ‘Information about antecedents’	1	Carney et al. (2012): ‘Module 3 presented information on the possible impact of medical malpractice concerns on recall rates’ [20]	0	N/A
	4.4 ‘Behavioural experiments’	2	von Lengerke et al. (2019): ‘4.4 Behavioural experiments, e.g. Fluorescence behaviour training by fluorescence methods using ultraviolet light boxes’ [54]	0	N/A
*5. Natural consequences*	5.1 ‘Information about health consequences’	66	Brunette et al. (2015): ‘Topics included facts about nicotine dependence in people with mental illness, nicotine withdrawal’ [69]	11	Abgrall et al. (2015): ‘We sent to all participating centers (including those in the simple information arm) a letter reminding them of the importance of adequate CKD management in people living with HIV’ [70]
			Bregnhøj et al. (2009): ‘The meeting included background information on the causes and consequences of polypharmacy, areas of concern in the treatment of the elderly and group discussions on patient cases’ [58]		
	5.2 ‘Salience of consequences’	3	Hallsworth et al. (2016): ‘Public health catastrophe’ [71]	1	Hallsworth et al. (2016): ‘Public health catastrophe’ [71]
			Soleymani et al. (2019): ‘The first page expresses the purpose of providing feedback and interpreting the colors used in the report and the importance of improving the situation of irrational prescribing signed by secretary of the committee’ [72]		
	5.3 ‘Information about social and environmental consequences’	18	Balas et al. (1998): ‘The clinical direct reports combine center specific information or practice patterns with the latest published evidence on the efficacy and cost of various dialysis modalities’ [73]	5	von Lengerke et al. (2019): ‘5.3 Information about social and environmental consequences, e.g. knowledge transfer on economic consequences of Nis’ [54]
	5.6 ‘Information about emotional consequences’	1	von Lengerke et al. (2019): ‘5.6 Information about emotional consequences, e.g. knowledge transfer on psychological consequences of Nis’ [54]	1	von Lengerke et al. (2019): ‘5.6 Information about emotional consequences, e.g. knowledge transfer on psychological consequences of Nis’ [54]
*6. Comparison of behaviour*	6.1 ‘Demonstration of the behaviour’	21	Gilkey et al. (2019): ‘…used video vignettes to demonstrate strategies for communicating with parents about HPV vaccination’ [74]	4	Buntinx et al. (1993): ‘During this period all 183 study doctors were provided with a copy of an article with photographs on the correct technique for obtaining cervical smears with different instruments’ [75]
			Kaminski et al. (2016): ‘Demonstration of colonoscopy withdrawal videos (inappropriate fold inspection, inadequate bowel distension, inappropriate suctioning technique)’ [76]		
	6.2 ‘Social comparison’	190	Ayieko et al. (2019): ‘Hospital performance in comparison with anonymized performance information of other hospitals’ [60]	40	Ayieko et al. (2019): ‘Each hospital’s performance compared to its own performance in the preceding period and also compared anonymously to other network hospitals’ [60]
	6.3 ‘Information about others’ approval’	3	von Lengerke et al. (2019): ‘6.3 Information about others’ approval, e.g. reflection of perceived recognition by superiors for compliance as assessed in survey’ [54]	1	Hallsworth et al. (2016): ‘Many practices are already taking action…’ [71]
*7. Associations*	7.1 ‘Prompts/cues’	83	Hocking et al. (2018): ‘A computer alert prompting testing of eligible patients’ [77]	14	Pape et al. (2011): ‘Automated DM-related point-of-care prompts’ [78]
*8. Repetition and substitution*	8.1 ‘Behavioural practice/rehearsal’	9	Cundill et al. (2015): ‘The final module was aimed at sustaining the change in practice by using challenging role-plays to practice integration of RDTs and demonstrate the capacity to problem solve a RDT logistical challenge’ [79]	2	Harris et al. (2013): ‘Hands-on experience with an insulin pen’ [56]
	8.2 ‘Behaviour substitution’	2	Hürlimann et al. (2015): ‘The main focus of the guidelines was to restrict prescriptions to bacterial infections and to preferentially prescribe narrow-spectrum antibiotics, namely penicillins for RTIs and trimethoprim/sulfamethoxazole for uncomplicated lower UTIs’ [80]	0	N/A
	8.6 ‘Generalisation of target behaviour’	1	von Lengerke et al. (2019): ‘8.6 Generalisation of target behaviour, e.g. transfer of problem-solving approaches across indications’ [54]	1	von Lengerke et al. (2019): ‘8.6 Generalisation of target behaviour, e.g. transfer of problem-solving approaches across indications’ [54]
	8.7 ‘Graded tasks’	4	von Lengerke et al. (2019): ‘8.7 Graded tasks, e.g. focusing on individual indications such as before aseptic procedures’ [54]	0	N/A
*9. Comparison of outcomes*	9.1 ‘Credible source’	149	Chaillet et al. (2015): ‘… provided by certified instructors from the Society of Obstetricians and Gynaecologists of Canada’ [81]	34	Wells et al. (2000): ‘For usual care, clinic medical directors were mailed the Agency for Healthcare Research and Quality depression practice guidelines, with quick reference guides for clinicians’ [82]
	9.2 ‘Pros and cons’	1	von Lengerke et al. (2019): ‘9.2 Pros and cons, e.g. discussing effects of compliance and noncompliance’ [54]	1	von Lengerke et al. (2019): ‘9.2 Pros and cons, e.g. discussing effects of compliance and noncompliance’ [54]
*10. Reward and threat*	10.1 ‘Material incentive (behaviour)’	7	Petersen et al. (2013): ‘Intervention group participants received up to five incentive payments in their paychecks approximately every four months and were notified each time a payment was posted’ [83]	1	Navathe et al. (2020): ‘The third column presents in blue the number of dollars earned per measure’ [84]
	10.2 ‘Material reward (behaviour)’	7	Fairbrother et al. (1999): ‘Physicians assigned to the bonus and feedback group were eligible to receive financial bonuses based on patients' up-to-date coverage for DTP and Haemophilus influenzae type b (Hib), OPV, and MMR’ [85]	0	N/A
	10.4 ‘Social reward’	6	Fuller et al. (2012): ‘If compliance was 100%, the staff member was praised’ [86]	1	Houston et al. (2015): ‘For ongoing facilitation, our study team completed a total of six proactive booster facilitation calls (approximately 15–30 min)… reinforcing success’ [87]
	10.5 ‘Social incentive’	2	Mertens et al. (2015): ‘Incentives to conduct SBIRT were limited to clinic recognition in the quality feedback reports (see “quality feedback reports” above) for high performing clinics’ [61]	0	N/A
	10.8 ‘Incentive (outcome)’	1	Navathe et al. (2020): ‘Primary care providers were each eligible for $75 three and six months after enrollment in the program if the patient’s hemoglobin A1c went down by at least 0.5 points from baseline or achieved a value of 9.0 or lower’ [84]	0	N/A
*12. Antecedents*	12.1 ‘Restructuring the physical environment’	7	Bonds et al. (2009): ‘…making structural changes to the clinic to improve blood pressure control’ [88]	1	Huis et al. (2013): ‘Facilities and products—screening and if necessary, adapt products and appropriate facilities [relating to hand hygiene]’ [89]
	12.2 ‘Restructuring the social environment’	69	Baldwin et al. (2010): ‘Selected staff from each intervention home were designated as infection control link workers, their role being to reinforce all aspects of good infection control throughout the study’ [90]	9	Lakshminarayan et al. (2010): ‘Clinical opinion leader recruitment in all hospitals’ [91]
	12.5 ‘Adding objects to the environment’	59	Kennedy et al. (2015): ‘Process Indicator Checklist: This tool assists teams with creating internal processes and policies that support and sustain appropriate prescribing and other osteoporosis and fractures best practices (e.g., admission/quarterly assessment, diagnoses documentation, ongoing staff education and training)’ [35]	10	Kennedy et al. (2015): ‘The tool-kit includes: the 10-min DVD (“Meeting the Challenge of Osteoporosis and Fracture Prevention”), informational pocket cards, case studies, and posters’ [35]
*13. Identity*	13.1 ‘Identification of self as role model’	1	von Lengerke et al. (2019): ‘13.1 Identification of self as role model, e.g. illustration and discussion of the function of role models in hand hygiene compliance’ [54]	0	N/A
	13.2 ‘Framing/reframing’	1	von Lengerke et al. (2019): ‘13.2 Framing/Reframing, e.g. raising the issue of compliance as a team task (team cooperation)’ [54]	0	N/A
*14. Scheduled consequences*	14.6 ‘Situation-specific reward’	1	von Lengerke et al. (2019): ‘14.6 Situation-specific reward, e.g. certification of ward with highest compliance with the trial’ [54]	0	N/A
*15. Self-belief*	15.1 ‘Verbal persuasion about capability’	1	von Lengerke et al. (2019): ‘15.1 Verbal persuasion about capability, e.g. discussion of positive compliance development’ [54]	1	von Lengerke et al. (2019): ‘15.1 Verbal persuasion about capability, e.g. discussion of positive compliance development’ [54]
	15.3 ‘Focus on past success’	1	von Lengerke et al. (2019): ‘15.3 Focus on past success, e.g. discussion of best year’ [54]	1	von Lengerke et al. (2019): ‘15.3 Focus on past success, e.g. discussion of best year’ [54]

^a^BCT not listed in the BCTTv1 but generated during our coding process. Numbers shown in first two columns relate to the numbering of BCT clusters and individual BCTs in the BCTTv1. N/A, BCT not identified

**Fig. 2:**
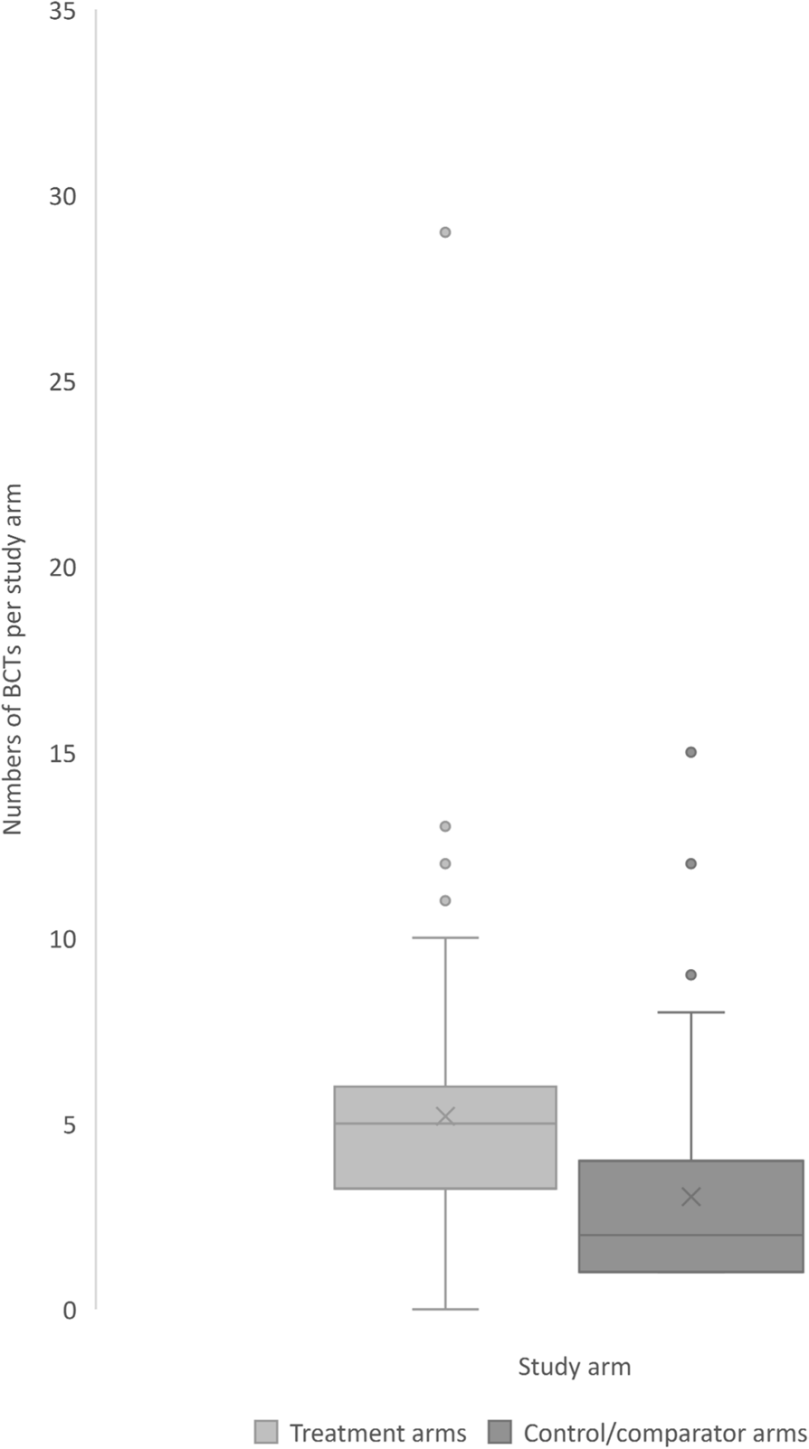
Box and whisker plot showing a comparison of BCT frequencies between treatment arms (*N* = 360) and control/comparator arms (*N* = 130 with at least one BCT present)

The median number of BCTs per A&F treatment arm was 5.0 (*IQR* = 2.3), and the total number of BCTs ranged from 1 to 29 BCTs in a single treatment arm (Fig. 2 shows a box and whisker plot of these data). Only 4/360 treatment arms (1%) included the BCTs ‘Feedback on behaviour’ without the presence of additional BCTs. The ten most frequently coded BCTs in A&F interventions evaluated in the trial treatment arms were as follows: (1) ‘Feedback on behaviour’ (320/360, 89%; e.g. providing feedback on drug prescribing); (2) ‘Instruction on how to perform the behaviour’ (255/360, 71%; e.g. issuing a clinical guideline to staff); (3) ‘Social comparison’ (190/360, 52%; e.g. providing feedback on the performance of peers); (4) ‘Credible source’ (149/360, 41%; e.g. endorsements from a respected professional body); (5) ‘Education (unspecified)’ (112/360, 31%; e.g. giving a lecture to staff); (6) ‘Social support (practical)’ (88/360, 24%; e.g. providing support to staff to achieve the target behaviour or outcome); (7) ‘Prompts/cues’ (83/360, 23%; e.g. a computer prompt when ordering a routine test); (8) ‘Problem-solving’ (76/360, 21%; e.g. identifying barriers and generating solutions towards achieving an audit standard); (9) ‘Discrepancy between current behaviour and goal’ (74/360, 21%; e.g. contrasting healthcare performance with a standard); and (10) ‘Restructuring the social environment’ (69/360, 19%; e.g. team member taking on an additional role to support practice change).

**Fig. 3:**
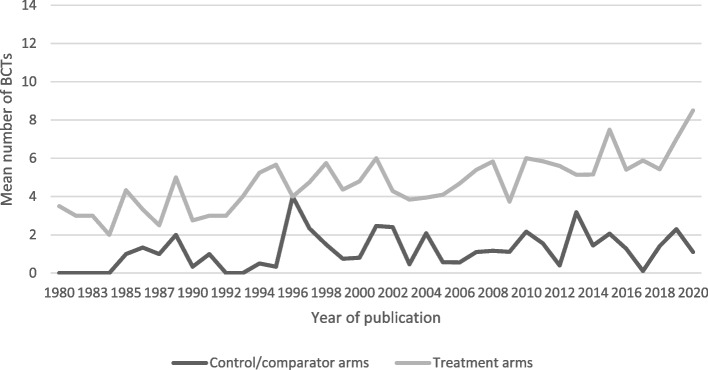
Mean BCT counts comparing treatment arms and control/comparator arms. Notes: Final search was done in June 2020, so 2020 data does not represent a full calendar year

Figure 3 shows our descriptive analysis of BCTs present in treatment arms by study publication year. There appears to be a trend for more BCTs to be reported in intervention descriptions for more recently published trials. Additionally, there appears to be an upwards trend in the maximum number of BCTs used in the last 5 years. The maximum number of BCTs used in a single study (*n* = 29 BCTs) was published within the last 5 years (see Appendix 3 for descriptive statistics for BCT frequencies by year).

### *Control/comparator arms (N* = *287)*

Table 3 lists the BCTs identified in control/comparator arms, their frequencies, and a corresponding example of how that BCT was operationalized in an A&F trial context. The most frequently coded BCTs (≥ 5 instances within treatment arms) are depicted in Fig. 1. In summary, a total of 131/287 (45%) control/comparator arms included at least one BCT. No BCTs were identified in 159/287 (55%) of control/comparator trial arms. Overall, 35 out of a possible 95 BCTs (37%) were identified in the comparator/control arm of at least one A&F trial. At least one BCT was identified in 14/16 possible clusters of BCTs. No BCTs were identified for the clusters: *regulation and covert learning.*

Excluding control/comparator arms with no BCTs (157/287; 55%), the median number of BCTs in 130/287 (45%) control/comparator arms was 2.0 (*IQR* = 3.0; range 0–15) (Fig. 2). Across all 287 control/comparator arms, the mean number of BCTs was 1.4 per control/comparator arm (*SD* = 2.2; median value for all 287 arms was 0.0, *IQR* = 2.0). The five most common BCTs reported in control/comparator arms were identical to those listed above in the treatment arms: (1) ‘Instruction on how to perform the behaviour’ (77/287; 27%), (2) ‘Feedback on behaviour’ (67/287, 23%), (3) ‘Social comparison’ (40/287; 14%), (4) ‘Credible source’ (34/287; 12%), and (5) ‘Education (unspecified)’ (28/287, 10%). There was a single instance of a BCTs that was present in control/comparator arms but not present in any of the treatment arms (‘Monitoring of outcome(s) of behaviour without feedback’).

Figure 3 shows our descriptive analysis of BCTs present in control/comparator arms by study publication year. There appears to be minimal change in the average number of BCTs over time. However, similar to the treatment arms, the maximum number of BCTs used in a single study (*n* = 15 BCTs) was published within the last 5 years (see Appendix 3 for descriptive statistics for BCT frequencies by year).

## Discussion

We sought to specify and synthesize the behaviour change content from 287 RCTs included in the ongoing update of a Cochrane systematic review of A&F interventions targeting healthcare professional practice change. We found that almost half of the BCTs from the 93-item BCTTv1 (plus two additional BCTs generated during the coding process; 95 BCTs in total) were identified at least once within treatment arms, and approximately a third of BCTs were present at least once within control/comparator arms.

Whilst a wide range of potential BCTs from the BCTTv1 were used, there are still many that remain unexplored (i.e. 50% from the original BCTTv1) and therefore could be targeted for future interventions targeting specific barriers to practice change among healthcare professionals. Although it is unlikely that all BCTs could be viably operationalized within A&F interventions targeting practice change (BCTs under consideration should be matched to known barriers and contextualized [92]), our analysis may help those looking to generate hypotheses, elucidate mechanisms of action, and identify unexplored components of A&F interventions, which should at a minimum be considered for their viability and appropriateness to the A&F context.

Multifaceted and multicomponent interventions (those containing multiple BCTs) were standard, which follows contemporary guidelines for delivering A&F for practice change [93]. Although the average number of BCTs within treatment arms was moderate (5.2 BCTs per arm, *SD* = 2.8), we know from the behaviour change literature that more active content within behavioural interventions does not always necessarily equal greater effectiveness [94, 95], and as such, further exploration of this is needed in the context of A&F. Furthermore, we know that the active content of interventions is often underreported, especially in less recent trials, and that the type of BCTs may be more important than the number per se when BCTs are matched to specific barriers/gaps for improving clinical practice [92].

The value of findings reported here is that it provides a methodologically rigorous dataset (and corresponding coding framework—see Appendix 2) for future analyses to answer questions such as which theory-based combinations of BCTs are used and how are BCTs or combinations linked to effectiveness (e.g. theory testing). Indeed, a number of behaviour change theories have been linked to A&F to try and explain the mechanisms through which A&F may bring about change in practice (e.g. control theory) [96]. Indeed, Gardner and colleagues proposed that three BCTs are linked to the key processes described in control theory: ‘Feedback on behaviour’, ‘Goal setting (behaviour)’, and ‘Action planning’. A&F interventions often occur as part of a more complex, multifaceted intervention and therefore may contain a much broader range of BCTs beyond these three previously linked to control theory. Future research is needed to assess how currently used BCTs align with mechanisms from other theories linked to A&F and which active ingredients in which combination help to explain effects. This in turn can lead to more focused suggestions for BCTs to include in the design and delivery of A&F trials. For example, our dataset will allow one to explore whether the presence of BCTs addressing the full range of constructs in control theory have larger treatment effects. Such questions are the focus of ongoing analyses being conducted as part of the Cochrane systematic review update and will be reported in future papers [18]. Additionally, there is always an opportunity to investigate the active ingredients of interventions at a more granular level. For example, there are likely to be BCTs embedded within actual feedback reports which would have been missed if an example was not provided or adequately described in published source materials. Although this level of detail was beyond the scope of our analysis, future research could examine some of the intervention materials provided to tease out additional BCTs and further operationalize BCTs in the A&F context. We therefore call for A&F trialists to provide examples of their A&F materials wherever possible to facilitate this level of synthesis.

We found that similar BCTs were consistently being included in both treatment and control/comparator arms. The most frequently identified BCTs in both treatment and control/comparator arms included providing behavioural feedback, sharing clinical guidelines, utilizing peer comparison data, providing education, and leveraging endorsements from credible sources (e.g. individuals, groups, organizations). Control/comparator arms having a large number of component BCTs, and thus potentially active behaviour change content, has been recognized as an consideration in trials of behaviour change interventions in other domains such as smoking cessation [12]. In certain cases, active controls differed from treatment arms in how the BCTs were delivered (i.e. mode of delivery) and/or frequency of delivery rather than the intervention content itself (often seen in head-to-head trials), which has been previously advocated for (e.g. more head-to-head trials comparing variants of A&F) [3, 97]. As it stands, the current version of the BCTTv1 does not account from dose, intensity, or operationalization of BCTs which may be an area to consider in future iterations of the taxonomy [98] and other behaviour change ontologies [99].

We found that education programmes were often poorly reported to the point where we decided to create a BCT—‘Education (unspecified)’—to capture relevant intervention content. Rather than creating new BCTs, other review studies have circumvented this issue by creating a coding assumptions for educational components of behaviour change interventions [94, 100] (coding the following two BCTs as a minimum to describe educational content: ‘Instruction on how to perform the behaviour’ and ‘Information about health consequences’). Providing information to increase knowledge was often central to A&F interventions, although knowledge alone may not be sufficient for lasting and meaningful behaviour change. Underreporting of the content of behaviour change interventions is a recognized, wider systemic challenge and limitation to these types of analyses [101, 102]. We have developed a coding framework containing BCTs operationalized in the A&F context. We believe that this will be a useful tool for intervention developers working in the field of A&F to help identify examples from the literature and consider how BCTs could be operationalized and incorporated into A&F intervention packages in both research and practice.

We found an apparent trend for more BCTs captured in intervention descriptions of more recently published trials, in line with our hypothesis. However, our findings should only be considered indicative as this may be an artefact of better reporting than evidence of the incorporation of more BCTs within A&F trials over time. A more comprehensive analysis of these data is required. Incidentally, the A&F trial that included the most BCTs (*n* = 29) was published in 2019 (von Lengerke, 2019) and was one of the few trials to use the BCTTv1 to describe the components of their A&F intervention, which vastly facilitated BCT coding and should be encouraged moving forward.

One of the benefits of using frameworks such as the BCTTv1 or similar taxonomies (e.g. (Effective Practice and Organisation of Care (EPOC)) [2] is that they help provide a shared language and definitions which should facilitate better reporting and design of behaviour change interventions. To date, the BCCTv1 has been used to code intervention content in a range of fields, predominantly focusing on patient and general population behaviours [11–13] with comparatively few instances of interventions targeting healthcare professional behaviour change [14, 19]. To our knowledge, this is the first time that the BCTTv1 has been comprehensively applied to the A&F literature. The application of behavioural science frameworks such as the BCTTv1 to different contexts can be a challenging process. Even among experienced researchers involved in this project, contextualizing BCTs within the A&F field came with its difficulties. As such, the coding framework we have developed should be viewed as a first iteration which will require further adaptation and refinement with the end goal of informing easy-to-use tools to facilitate better reporting and design of A&F interventions.

Previous work by Colquhoun and colleagues involved a prioritization exercise with A&F experts to generate hypotheses most likely to advance the field [103]. Seven hypotheses were chosen by > 50% of respondents and included the statements ‘A&F interventions would be more effective if feedback was provided by a trusted source’ and ‘if it suggests clear action plans’. Both these hypotheses map directly onto two BCTs listed in the BCTTv1, ‘Credible source’ and ‘Action planning’, respectively. ‘Credible source’ was a frequently coded BCT (present in 41% of treatment arms); however, ‘Action planning’ was not (present in 8% of treatment arms). The recommendation for specific corrective actions (e.g. recipient-generated if–then plans) alongside the feedback has been suggested previously [93], yet this remains absent in > 90% of trials of A&F interventions. As such, further exploration about how to best optimize A&F interventions is warranted and should be supported using rigorous evidence synthesis methods coupled with consensus building exercises among A&F experts.

A&F is often deployed as part of multi-level, organizational-level, and quality improvement interventions which may address barriers at the healthcare professional, healthcare team, or health system level. There is an argument whether the BCTTv1 is sensitive enough to capture key content at these various levels of change. In particular, system-level strategies (e.g. changes to care pathways) do not always include the necessary depth of detail needed to be captured by the BCTTv1 but may be more easily accounted for in other taxonomies such as EPOC [2] (for which A&F is incidentally listed as intervention subcategory within EPOC). However, some system-level strategies such as ‘Restructuring the social environment’ were frequently coded meaning that the BCTTv1 was sensitive enough to pick up some broader-level BCTs.

### Limitations

First, our findings are limited by underreporting of intervention content in A&F trials (i.e. we suspect more BCTs were present in interventions than the number found in our results); however, the increased use of reporting checklists such as TIDIeR should help with transparency in more recently published trials [16]. Second, our BCT analysis related to whether each BCT was ‘present’ or ‘absent’ and did not account for BCT dose, frequency, or operationalization (e.g. different forms of providing A&F graphs or charts to providers) which could moderate the effectiveness of A&F interventions. Moreover, we did not distinguish whether BCTs were attributed to A&F components specifically or whether they were part of co-intervention components which limit the extent to which we can link specific BCTs directly to specific A&F components. Our current analysis also did not account for the extent to which BCTs were implemented with consideration to the context in which they were delivered and the theory underpinning their mechanisms of action. Whilst this was outside of the scope of this paper, identifying which studies incorporated logic models into their design to inform the selection of intervention components would be an important consideration to help understand why some BCTs are effective or not in different contexts [3]. Third, the BCTs ‘Education (unspecified)’ and ‘Feedback (unspecified)’ are broad, nonspecific codes which helped capture studies with limited descriptions of the intervention components. Whilst we believe this was warranted for the purposes of our analyses, the addition of broad, nonspecific BCTs provides little understanding about the context of their use and thus should be limited for future use. Fourth, it was sometimes difficult to tease apart what constituted the active ingredients of the A&F intervention itself (within the scope of our analysis) and the implementation strategies delivered alongside the intervention (outside of the scope of our analysis) which may have resulted in some inconsistent coding. Better reporting in this area would improve this issue moving forward. An interesting line for further investigation would be to explore the range and types of implementation processes used in conjunction with A&F interventions. Fifth, the term benchmarking has been defined as a social comparison derived from the performance of a population, to draw attention to the performance of others [104]. For our analysis, we chose to capture ‘benchmarking’ (when a healthcare provider behaviour was contrasted with a ‘standard’) as the BCT ‘Discrepancy between current behaviour and goal’, rather than as a social comparison. If targets/feedback incorporated peer-driven data as opposed to a ‘standard’, we captured as the BCT ‘Social comparison’. A national benchmark may serve as a standard or be adopted as a goal depending on the context in which it is delivered, and as such, greater clarity on definitions for the content of performance information is likely needed. Sixth, our current analysis of BCTs over time was descriptive and thus limited. More formal analysis such as an exploratory time series analysis was outside the scope of the paper; however, further inquiry into this relationship is warranted.

## Conclusions

We have identified the number and type of BCTs used within 287 RCTs of A&F interventions, one of the largest analyses of its kind. We have developed a coding framework to help operationalize BCTs in the A&F context and believe this to be an important step for higher-quality reporting and to facilitate subsequent optimization, reproduction, scaling, and spread of effective versions of A&F.

### Supplementary Information

**Additional file 1: Appendix 1. **List of 287 included studies.

**Additional file 2: Appendix 2. **Coding framework for behaviour change techniques, operationalized for audit and feedback interventions.

**Additional file 3: Appendix 3. **Descriptive statistics for BCT frequencies by study year of publication.

## Data Availability

The datasets used and/or analysed during the current study are available from the corresponding author on reasonable request.

## Abbreviations

A&F: Audit and feedback
BCT: Behaviour change technique
BCTTv1: Behaviour Change Technique Taxonomy Version 1
EPOC: Effective Practice and Organisation of Care
TIDIeR: Template for Intervention Description and Replication
