# Correction: Identifying behaviour change techniques in 287 randomized controlled trials of audit and feedback interventions targeting practice change among healthcare professionals

**DOI:** 10.1186/s13012-023-01328-6

**Published:** 2023-12-18

**Authors:** Jacob Crawshaw, Carly Meyer, Vivi Antonopoulou, Jesmin Antony, Jeremy M. Grimshaw, Noah Ivers, Kristin Konnyu, Meagan Lacroix, Justin Presseau, Michelle Simeoni, Sharlini Yogasingam, Fabiana Lorencatto

**Affiliations:** 1 Centre for Evidence-Based Implementation, Hamilton Health Sciences, Hamilton, ON Canada; 2 Department of Medicine, McMaster University, Hamilton, ON Canada; 3 Department of Clinical, Educational and Health Psychology, Centre for Behaviour Change, University College London, London, WC1E 7HB UK; 4 NIHR Policy Research Unit in Behavioural Science, Newcastle University, Baddiley-Clark Building, Richardson Road, Newcastle Upon Tyne, NE2 4AX UK; 5 Women’s College Research Institute, Women’s College Hospital, Toronto, ON Canada; 6 Centre for Implementation Research, Ottawa Hospital Research Institute, Ottawa, Canada; 7 School of Epidemiology and Public Health, University of Ottawa, Ottawa, Canada; 8 Department of Health Services, Policy and Practice, Center for Evidence Synthesis in Health, Brown University School of Public Health, Brown University, Providence, Rhode Island USA

**Correction: Implement Sci 18, 63 (2023)**

**https://doi.org/10.1186/s13012-023-01318-8**

After the publication of the original article [1], the authors reported errors in the typesetting of value mentioned in Abstract. In the Result section of Abstract, it is written that ‘range = 129 per arm’. It should have been written ‘range = 1-29’.

The original article [1] has been updated.
