# Supplementary material for: Identifying behaviour change techniques in 287 randomized controlled trials of audit and feedback interventions targeting practice change among healthcare professionals
Source: Implement Sci. 2023 Nov 21;18:63. doi: 10.1186/s13012-023-01318-8 (PMC10664600; doi:10.1186/s13012-023-01318-8)
Supplement: Supplementary file 1 — Additional file 1: Appendix 1. List of 287 included studies. [file 13012_2023_1318_MOESM1_ESM.docx]

***Appendix 1* – List of 287 included studies**

1. Allison JJ, Kiefe CI, Wall T, Casebeer L, Ray MN, Spettell CM, et al. Multicomponent Internet continuing medical education to promote chlamydia screening. Am J Prev Med. 2005;28(3):285-90.

2. Althabe F, Buekens P, Bergel E, Belizán José M, Campbell Marci K, Moss N, et al. A Behavioral Intervention to Improve Obstetrical Care. N Engl J Med. 2008;358(18):1929-40.

3. Althabe F, Chomba E, Tshefu AK, Banda E, Belizan M, Bergel E, et al. A multifaceted intervention to improve syphilis screening and treatment in pregnant women in Kinshasa, Democratic Republic of the Congo and in Lusaka, Zambia: a cluster randomised controlled trial. Lancet Glob Health. 2019;7(5):e655-e63.

4. Amanyire G, Semitala FC, Namusobya J, Katuramu R, Kampiire L, Wallenta J, et al. Effects of a multicomponent intervention to streamline initiation of antiretroviral therapy in Africa: a stepped-wedge cluster-randomised trial. Lancet HIV. 2016;3(11):e539-e48.

5. Anderson FA, Jr., Wheeler HB, Goldberg RJ, Hosmer DW, Forcier A, Patwardhan NA. Changing clinical practice. Prospective study of the impact of continuing medical education and quality assurance programs on use of prophylaxis for venous thromboembolism. AMA Arch Intern Med. 1994;154:669-77.

6. Aspy CB, Enright M, Halstead L, Mold JW. Improving mammography screening using best practices and practice enhancement assistants: an Oklahoma Physicians Resource/Research Network (OKPRN) study. J Am Board Fam Med. 2008;21(4):326-33.

7. Avery AJ, Rodgers S, Cantrill JA, Armstrong S, Cresswell K, Eden M, et al. A pharmacist-led information technology intervention for medication errors (PINCER): a multicentre, cluster randomised, controlled trial and cost-effectiveness analysis. Lancet. 2012;379(9823):1310-9.

8. Awad AI, Eltayeb IB, Baraka OZ, Cano-Garcinuno A, az-Vazquez C, Carvajal-Uruena I. Changing antibiotics prescribing practices in health centers of Khartoum State, Sudan; Group education on asthma for children and caregivers: a randomized, controlled trial addressing effects on morbidity and quality of life. Eur J Clin Pharmacol. 2006;62:135-42.

9. Ayieko P, Ntoburi S, Wagai J, Opondo C, Opiyo N, Migiro S, et al. A multifaceted intervention to implement guidelines and improve admission paediatric care in Kenyan district hospitals: a cluster randomised trial. PLoS Med. 2011;8(4):e1001018.

10. Ayieko P, Irimu G, Ogero M, Mwaniki P, Malla L, Julius T, et al. Effect of enhancing audit and feedback on uptake of childhood pneumonia treatment policy in hospitals that are part of a clinical network: a cluster randomized trial. Implement Sci. 2019;14(1):20.

11. Bahrami M, Deery C, Clarkson JE, Pitts NB, Johnston M, Ricketts I. Effectiveness of strategies to disseminate and implement clinical guidelines for the management of impacted and unerupted third molars in primary dental care, a cluster randomised controlled trial. Br Dent J. 2004;197(11):691-6.

12. Baker R, Farooqui A, Tait C, Walsh S. Randomised controlled trial of reminders to enhance the impact of audit in general practice on management of patients who use benzodiazepines. Qual Health Care. 1997;6:14-8.

13. Baker R, Falconer J, Lambert PC. Randomized controlled trial of the effectiveness of feedback in improving test ordering in general practice. Scand J Prim Health Care. 2003;21:219-23.

14. Baker R, Falconer J, Lambert PC. Randomized controlled trial of the effectiveness of feedback in improving test ordering in general practice. Scand J Prim Health Care. 2003;21:219-23.

15. Baker R, Fraser RC, Stone M, Lambert P, Stevenson K, Shiels C. Randomised controlled trial of the impact of guidelines, prioritised review criteria and feedback on implementation of recommendations for angina and asthma. Br J Gen Pract. 2003;53:284-91.

16. Baker R, Fraser RC, Stone M, Lambert P, Stevenson K, Shiels C. Randomised controlled trial of the impact of guidelines, prioritised review criteria and feedback on implementation of recommendations for angina and asthma. Br J Gen Pract. 2003;53:284-91.

17. Balas E, Boren SA, Hicks LL, Chonko AM, Stephenson K. Effect of linking practice data to published evidence: A randomized controlled trial of clinical direct reports. Med Care. 1998;36:79-87.

18. Baldwin NS, Gilpin DF, Tunney MM, Kearney MP, Crymble L, Cardwell C, et al. Cluster randomised controlled trial of an infection control education and training intervention programme focusing on meticillin-resistant Staphylococcus aureus in nursing homes for older people. J Hosp Infect. 2010;76(1):36‐41.

19. Barkun AN, Bhat M, Armstrong D, Dawes M, Donner A, Enns R, et al. Effectiveness of disseminating consensus management recommendations for ulcer bleeding: a cluster randomized trial. CMAJ. 2013;185(3):E156-66.

20. Batty G, Hooper R, Oborne CA, Jackson S. Investigating intervention strategies to increase the appropriate use of anti-thrombotics in elderly hospital in-patients with atrial fibrillation. Br J Clin Gov. 2001;6(4):252-58.

21. Beck CA, Richard H, Tu JV, Pilote L. Administrative Data Feedback for Effective Cardiac Treatment: AFFECT, a cluster randomized trial. JAMA. 2005;294:309-17.

22. Beeckman D, Clays E, Van Hecke A, Vanderwee K, Schoonhoven L, Verhaeghe S. A multi-faceted tailored strategy to implement an electronic clinical decision support system for pressure ulcer prevention in nursing homes: a two-armed randomized controlled trial. Int J Nurs Stud. 2013;50(4):475-86.

23. Belcher DW. Implementing preventive services. Success and failure in an outpatient trial. Arch Intern Med. 1990;150(12):2533-41.

24. Bentz CJ, Bayley KB, Bonin KE, Fleming L, Hollis JF, Hunt JS. Provider feedback to improve 5A's tobacco cessation in primary care: a cluster randomized clinical trial. Nicotine Tob Res. 2007;9(3):341-9.

25. Bertoni AG, Bonds DE, Chen H, Hogan P, Crago L, Rosenberger E, et al. Impact of a multifaceted intervention on cholesterol management in primary care practices: guideline adherence for heart health randomized trial. Arch Intern Med. 2009;169(7):678-86.

26. Bhatia RS, Dudzinski DM, Malhotra R, Milford CE, Yoerger Sanborn DM, Picard MH, et al. Educational intervention to reduce outpatient inappropriate echocardiograms: a randomized control trial. JACC Cardiovasc Imaging. 2014;7(9):857-66.

27. Bhatia RS, Ivers NM, Yin XC, Myers D, Nesbitt GC, Edwards J, et al. Improving the Appropriate Use of Transthoracic Echocardiography: The Echo WISELY Trial. J Am Coll Cardiol. 2017;70(9):1135-44.

28. Blais R, Laurier C, Paré M. Effect of feedback letters to physicians and pharmacists on the appropriate use of medication in the treatment of asthma. J Asthma. 2008;45(3):227-31.

29. Blais R, Laurier C, Paré M. Effect of feedback letters to physicians and pharmacists on the appropriate use of medication in the treatment of asthma. J Asthma 2008;45(3):227-31.

30. Bloos F, Ruddel H, Thomas-Ruddel D, Schwarzkopf D, Pausch C, Harbarth S, et al. Effect of a multifaceted educational intervention for anti-infectious measures on sepsis mortality: a cluster randomized trial. Intensive Care Med. 2017;43(11):1602-12.

31. Boekeloo BO, Becker DM, Levine DM, Belitsos PC, Pearson TA. Strategies for increasing house staff management of cholesterol with inpatients. Am J Prev Med. 1990;6(Suppl 2):51-9.

32. Boet S, Bryson GL, Taljaard M, Pigford AA, McIsaac DI, Brehaut J, et al. Effect of audit and feedback on physicians' intraoperative temperature management and patient outcomes: a three-arm cluster randomized-controlled trial comparing benchmarked and ranked feedback. Can J Anaesth. 2018;65(11):1196-209.

33. Bond TC, Patel PR, Krisher J, Sauls L, Deane J, Strott K, et al. A group-randomized evaluation of a quality improvement intervention to improve influenza vaccination rates in dialysis centers. Am J Kidney Dis. 2011;57(2):283-90.

34. Bonds DE, Hogan PE, Bertoni AG, Chen H, Clinch CR, Hiott AE, et al. A multifaceted intervention to improve blood pressure control: The Guideline Adherence for Heart Health (GLAD) study. Am Heart J. 2009;157(2):278-84.

35. Bonevski B, Sanson-Fisher RW, Campbell E, Carruthers A, Reid ALA, Ireland M. Randomized controlled trial of a computer strategy to increase general practitioner preventive care. Prev Med. 1999;29:478-86.

36. Borgiel AEM, Williams JI, Davis DA, Dunn EV, Hobbs N, Hutchison B. Evaluating the effectiveness of 2 educational interventions on family practice. CMAJ. 1999;8:965-70.

37. Brady WJ, Hissa DC, McConnell M, Wones RG. Should physicians perform their own quality assurance audits? J Gen Intern Med. 1988;3:560-5.

38. Brady WJ, Hissa DC, McConnell M, Wones RG. Should physicians perform their own quality assurance audits? J Gen Intern Med. 1988;3:560-5.

39. Bregnhøj L, Thirstrup S, Kristensen MB, Bjerrum L, Sonne J. Combined intervention programme reduces inappropriate prescribing in elderly patients exposed to polypharmacy in primary care. Eur J Clin Pharmacol. 2009;65(2):199-207.

40. Brown LF, Keily PA, Spencer AJ. Evaluation of a continuing education intervention "Periodontics in General Practice". Community Dent Oral Epidemiol. 1994;22:441-7.

41. Brown B, Young J, Smith DP, Kneebone AB, Brooks AJ, Egger S, et al. A multidisciplinary team-oriented intervention to increase guideline recommended care for high-risk prostate cancer: A stepped-wedge cluster randomised implementation trial. Implement Sci. 2018;13(1):43.

42. Brunette MF, Dzebisashvili N, Xie H, Akerman S, Ferron JC, Bartels S. Expanding Cessation Pharmacotherapy Via Videoconference Educational Outreach to Prescribers. Nicotine Tob Res. 2015;17(8):960-7.

43. Buffington J, Bell KM, LaForce FM. A target-based model for increasing influenza immunizations in private practice. J Gen Intern Med. 1991;6:204-9.

44. Campbell E, Walsh RA, Sanson-Fisher R, Burrows S, Stojanovski E. A group randomised trial of two methods for disseminating a smoking cessation programme to public antenatal clinics: effects on patient outcomes. Tob Control. 2006;15(2):97-102.

45. Cánovas JJ, Hernández PJ, Botella JJ. Effectiveness of internal quality assurance programmes in improving clinical practice and reducing costs. J Eval Clin Pract. 2009;15(5):813-9.

46. Carney PA, Abraham L, Cook A, Feig SA, Sickles EA, Miglioretti DL, et al. Impact of an Educational Intervention Designed to Reduce Unnecessary Recall during Screening Mammography. Acad Radiol. 2012;19(9):1114-20.

47. Chaillet N, Dumont A, Abrahamowicz M, Pasquier JC, Audibert F, Monnier P, et al. A cluster-randomized trial to reduce cesarean delivery rates in Quebec. N Engl J Med. 2015;372(18):1710-21.

48. Charrier L, Allochis MC, Cavallo MR, Gregori D, Cavallo F, Zotti CM. Integrated audit as a means to implement unit protocols: a randomized and controlled study. J Eval Clin Pract. 2008;14(5):847-53.

49. Chassin MR, McCue SM. A randomized trial of medical quality assurance. Improving physicians' use of pelvimetry. JAMA. 1986;256:1012-6.

50. Cheater FM, Baker R, Reddish S, Spiers N, Wailoo A, Gillies C. Cluster randomized controlled trial of the effectiveness of audit and feedback and educational outreach on improving nursing practice and patient outcomes. Med Care. 2006;44(6):542-51.

51. Clarke M, Devane D, Gross MM, Morano S, Lundgren I, Sinclair M, et al. OptiBIRTH: a cluster randomised trial of a complex intervention to increase vaginal birth after caesarean section. BMC Pregnancy Childbirth. 2020;20(1):143.

52. Clyne B, Smith SM, Hughes CM, Boland F, Bradley MC, Cooper JA, et al. Effectiveness of a Multifaceted Intervention for Potentially Inappropriate Prescribing in Older Patients in Primary Care: A Cluster-Randomized Controlled Trial (OPTI-SCRIPT Study). Ann Fam Med. 2015;13(6):545-53.

53. Colon-Emeric CS, Lyles KW, House P, Levine DA, Schenck AP, Allison J, et al. Randomized trial to improve fracture prevention in nursing home residents. Am J Med. 2007;120(10):886-92.

54. Coma E, Medina M, Mendez L, Hermosilla E, Iglesias M, Olmos C, et al. Effectiveness of electronic point-of-care reminders versus monthly feedback to improve adherence to 10 clinical recommendations in primary care: a cluster randomized clinical trial. BMC Med Inf Decis Mak. 2019;19(1):245.

55. Crotty M, Whitehead C, Rowett D, Halbert J, Weller D, Finucane P, et al. An outreach intervention to implement evidence based practice in residential care: a randomized controlled trial [ISRCTN67855475]. BMC Health Serv Res. 2004;4(1):6.

56. Cundill B, Mbakilwa H, Chandler CI, Mtove G, Mtei F, Willetts A, et al. Prescriber and patient-oriented behavioural interventions to improve use of malaria rapid diagnostic tests in Tanzania: facility-based cluster randomised trial. BMC Med. 2015;13:118.

57. Curtis JR, Olivieri J, Allison JJ, Gaffo A, Juarez L, Kovac SH. A group randomized trial to improve safe use of nonsteroidal anti-inflammatory drugs. Am J Manag Care. 2005;11(9):537-43.

58. Curtis JR, Westfall AO, Allison J, Becker A, Melton ME, Freeman A. Challenges in improving the quality of osteoporosis care for long-term glucocorticoid users: a prospective randomized trial. AMA Arch Intern Med. 2007;167(6):591-6.

59. Curtis JR, Nielsen EL, Treece PD, Downey L, Dotolo D, Shannon SE, et al. Effect of a quality-improvement intervention on end-of-life care in the intensive care unit: a randomized trial. Am J Respir Crit Care Med. 2011;183(3):348-55.

60. DeVore AD, Cox M, Heidenreich PA, Fonarow GC, Yancy CW, Eapen ZJ, et al. Cluster-Randomized Trial of Personalized Site Performance Feedback in Get With The Guidelines-Heart Failure. Circ Cardiovasc Qual Outcomes. 2015;8(4):421-7.

61. Diamantouros A, Kiss A, Papastavros T, U D, Zwarenstein M, Geerts WH. The TOronto ThromboProphylaxis Patient Safety Initiative (TOPPS): A cluster randomised trial. Res Social Adm Pharm. 2017;13(5):997-1003.

62. Dijkstra RF, Braspenning JC, Huijsmans Z, Akkermans RP, van Ballegooie E, ten Have P, et al. Introduction of diabetes passports involving both patients and professionals to improve hospital outpatient diabetes care. Diabetes Res Clin Pract. 2005;68(2):126-34.

63. Dormuth CR, Carney G, Taylor S, Bassett K, Maclure M. A randomized trial assessing the impact of a personal printed feedback portrait on statin prescribing in primary care. J Contin Educ Health Prof. 2012;32(3):153-62.

64. Dreischulte T, Donnan P, Grant A, Hapca A, McCowan C, Guthrie B. Safer Prescribing--A Trial of Education, Informatics, and Financial Incentives. N Engl J Med. 2016;374(11):1053-64.

65. Dudzinski DM, Bhatia RS, Mi MY, Isselbacher EM, Picard MH, Weiner RB. Effect of Educational Intervention on the Rate of Rarely Appropriate Outpatient Echocardiograms Ordered by Attending Academic Cardiologists: A Randomized Clinical Trial. JAMA Cardiol. 2016;1(7):805-12.

66. Eccles M, Steen N, Grimshaw J, Thomas L, McNamee P, Soutter J. Effect of audit and feedback, and reminder messages on primary-care radiology referrals: a randomised trial. Lancet. 2001;357(9266):1406-9.

67. Eltayeb IB, Awad AI, Mohamed-Salih MS, Daffa-Alla MA, Ahmed MB, Ogail MA. Changing the prescribing patterns of sexually transmitted infections in the White Nile Region of Sudan. Sex Transm Infect. 2005;81:426-7.

68. Estrada CA, Safford MM, Salanitro AH, Houston TK, Curry W, Williams JH, et al. A web-based diabetes intervention for physician: a cluster-randomized effectiveness trial. Int J Qual Health Care. 2011;23(6):682-9.

69. *Billue KL, Safford MM, Salanitro AH, Houston TK, Curry W, Kim Y, et al. Medication intensification in diabetes in rural primary care: a cluster-randomised effectiveness trial. BMJ Open. 2012;2(5).

70. Everett GD, deBlois CS, Chang PF, Holets T. Effect of cost education, cost audits, and faculty chart review on the use of laboratory services. AMA Arch Intern Med. 1983;143:942-4.

71. Fabbri C, Dutt V, Shukla V, Singh K, Shah N, Powell-Jackson T. The effect of report cards on the coverage of maternal and neonatal health care: a factorial, cluster-randomised controlled trial in Uttar Pradesh, India. Lancet Glob Health. 2019;7(8):e1097-e108.

72. Fairbrother G, Hanson KL, Friedman S, Butts GC. The impact of physician bonuses, enhanced fees, and feedback on childhood immunization coverage rates. Am J Public Health. 1999;89(2):171-5.

73. Feder G, Griffiths C, Highton C, Eldridge S, Spence M, Southgate L. Do clinical guidelines introduced with practice based education improve care of asthmatic and diabetic patients? A randomised controlled trial in general practices in east London. BMJ. 1995;311(7018):1473-8.

74. Feder G, Griffiths C, Highton C, Eldridge S, Spence M, Southgate L. Do clinical guidelines introduced with practice based education improve care of asthmatic and diabetic patients? A randomised controlled trial in general practices in east London. BMJ. 1995;311(7018):1473-8.

75. Ferguson TB, Peterson ED, Coombs LP, Eiken MC, Carey ML, Grover FL. Use of contiouous quality improvement to increase use of process measures in patients undergoing coronary artery bypass graft surgery. JAMA. 2003;290(1):49-56.

76. Ferguson TB, Peterson ED, Coombs LP, Eiken MC, Carey ML, Grover FL, et al. Use of contiouous quality improvement to increase use of process measures in patients undergoing coronary artery bypass graft surgery. JAMA 2003;290(1):49-56.

77. Fiks AG, Grundmeier RW, Mayne S, Song L, Feemster K, Karavite D, et al. Effectiveness of decision support for families, clinicians, or both on HPV vaccine receipt. Pediatrics. 2013;131(6):1114-24.

78. Fiks AG, Mayne SL, Michel JJ, Miller J, Abraham M, Suh A, et al. Distance-Learning, ADHD Quality Improvement in Primary Care: A Cluster-Randomized Trial. J Dev Behav Pediatr. 2017;38(8):573-83.

79. Filardo G, Nicewander D, Herrin J, Edwards J, Galimbertti P, Tietze M. A hospital-randomized controlled trial of a formal quality improvement educational program in rural and small community Texas hospitals: one year results. Int J Qual Health Care. 2009;21:225-32.

80. Finkelstein JA, Davis RL, Dowell SF, Metlay JP, Soumerai SB, Rifas-Shiman SL, et al. Reducing antibiotic use in children: a randomized trial in 12 practices. Pediatrics. 2001;108(1):1-7.

81. Foster JM, Hoskins G, Smith B, Lee AJ, Price D, Pinnock H. Practice development plans to improve the primary care management of acute asthma: randomised controlled trial. BMC Fam Pract. 2007;8:23.

82. Foy R, Penney GC, Grimshaw JM, Ramsay CR, Walker AE, MacLennan G. A randomised controlled trial of a tailored multifaceted strategy to promote implementation of a clinical guideline on induced abortion care. BJOG. 2004;111(7):726-33.

83. Fretheim A, Oxman AD, Havelsrud K, Treweek S, Kristoffersen DT, Bjorndal A. Rational prescribing in primary care (RaPP): a cluster randomized trial of a tailored intervention. PLoS Med. 2006;3(6):e134.

84. Frijling BD, Lobo CM, Hulscher MEJL, Akkarmans RP, Braspenning JCC, Prins A. Multifaceted support to improve clinical decision making in diabetes care: a randomized controlled trial in general practice. Diabet Med. 2002;19:836-42.

85. Frijling BD, Lobo CM, Hulscher MEJL, Akkarmans RP, Van Drenth BB, Prins A. Intensive support to improve clinical decision making in cardiovascular care: a randomised controlled trial in general practice. Qual Saf Health Care. 2003;12:181-7.

86. Fuller C, Michie S, Savage J, McAteer J, Besser S, Charlett A, et al. The Feedback Intervention Trial (FIT)--improving hand-hygiene compliance in UK healthcare workers: a stepped wedge cluster randomised controlled trial. PLoS ONE. 2012;7(10):e41617.

87. Ganz PA, Farmer MM, Belman MJ, Garcia CA, Streja L, Dietrich AJ, et al. Results of a randomized controlled trial to increase colorectal cancer screening in a managed care health plan. Cancer. 2005;104(10):2072-83.

88. Geary L, Hasselstrom J, Carlsson AC, Eriksson I, von Euler M. Secondary prevention after stroke/transient ischemic attack: A randomized audit and feedback trial. Acta Neurol Scand. 2019;140(2):107-15.

89. Gerber JS, Prasad PA, Fiks AG, Localio AR, Bell LM, Keren R, et al. Durability of benefits of an outpatient antimicrobial stewardship intervention after discontinuation of audit and feedback. JAMA. 2014;312(23):2569-70.

90. Gilkey MB, Dayton AM, Moss JL, Sparks AC, Grimshaw AH, Bowling JM, et al. Increasing provision of adolescent vaccines in primary care: a randomized controlled trial. Pediatrics. 2014;134(2):e346-53.

91. Gilkey MB, Parks MJ, Margolis MA, McRee AL, Terk JV. Implementing Evidence-Based Strategies to Improve HPV Vaccine Delivery. Pediatrics. 2019;144(1).

92. Gjelstad S, Hoye S, Straand J, Brekke M, Dalen I, Lindbaek M. Improving antibiotic prescribing in acute respiratory tract infections: cluster randomised trial from Norwegian general practice (prescription peer academic detailing (Rx-PAD) study). BMJ. 2013;347:f4403.

93. Goderis G, Borgermans L, Grol R, Van Den Broeke C, Boland B, Verbeke G, et al. Start improving the quality of care for people with type 2 diabetes through a general practice support program: a cluster randomized trial. Diabetes Res Clin Pract. 2010;88(1):56-64.

94. Goff DC, Gu L, Cantley LK, Sheedy DJ, Cohen SJ. Quality of care for secondary prevention for patients with coronary heart disease: Results of the hastening the effective application of research through technology (HEART) trial. Am Heart J. 2003;146(6):1045-151.

95. Goldberg HI, Wagner EH, Fihn SD, Martin DP, Horowitz CR, Christensen DB, et al. A randomized controlled trial of CQI teams and academic detailing: can they alter compliance with guidelines? Jt Comm J Qual Improv. 1998;24(3):130-42.

96. Gonzales R, Anderer T, McCulloch CE, Maselli JH, Bloom F, Graf TR, et al. A cluster randomized trial of decision support strategies for reducing antibiotic use in acute bronchitis. JAMA Intern Med. 2013;173(4):267-73.

97. Grady KE, Lemkau JP, Lee NR, Caddell C. Enhancing mammography referral in primary care. Prev Med. 1997;26:791-800.

98. Guadagnoli E, Soumerai SB, Gurwitz JH, Borbas C, Shapiro CL, Weeks JC. Improving discussion of surgical treatment options for patients with breast cancer: local medical opinion leaders versus audit and performance feedback. Breast Cancer Res Treat. 2000;61(2):171-5.

99. Gude WT, van Engen-Verheul MM, van der Veer SN, Kemps HM, Jaspers MW, de Keizer NF, et al. Effect of a web-based audit and feedback intervention with outreach visits on the clinical performance of multidisciplinary teams: a cluster-randomized trial in cardiac rehabilitation. Implement Sci. 2016;11(1):160.

100. Gude WT, van Engen-Verheul MM, van der Veer SN, Kemps HM, Jaspers MW, de Keizer NF, et al. Effect of a web-based audit and feedback intervention with outreach visits on the clinical performance of multidisciplinary teams: a cluster-randomized trial in cardiac rehabilitation. Implement Sci 2016;11(1):160.

101. Guldberg TL, Vedsted P, Kristensen JK, Lauritzen T. Improved quality of Type 2 diabetes care following electronic feedback of treatment status to general practitioners: a cluster randomized controlled trial. Diabet Med. 2011;28(3):325-32.

102. Gulliford MC, Juszczyk D, Prevost AT, Soames J, McDermott L, Sultana K, et al. Electronically delivered interventions to reduce antibiotic prescribing for respiratory infections in primary care: cluster RCT using electronic health records and cohort study. Health Technol Assess. 2019;23(11):1-70.

103. Gullion DS, Tschann JM, Adamson TE, Coates TJ. Management of hypertension in private practice: a randomized controlled trial in continuing medical education. J Contin Educ Health Prof. 1988;8:239-55.

104. Guthrie B, Kavanagh K, Robertson C, Barnett K, Treweek S, Petrie D, et al. Data feedback and behavioural change intervention to improve primary care prescribing safety (EFIPPS): multicentre, three arm, cluster randomised controlled trial. BMJ. 2016;354:i4079.

105. Hallsworth M, Chadborn T, Sallis A, Sanders M, Berry D, Greaves F, et al. Provision of social norm feedback to high prescribers of antibiotics in general practice: a pragmatic national randomised controlled trial. Lancet. 2016;387(10029):1743-52.

106. Harris SB, Gerstein HC, Yale JF, Berard L, Stewart J, Webster-Bogaert S, et al. Can community retail pharmacist and diabetes expert support facilitate insulin initiation by family physicians? Results of the AIM@GP randomized controlled trial. BMC Health Serv Res. 2013;13:71.

107. Harris MF, Parker SM, Litt J, van Driel M, Russell G, Mazza D, et al. Implementing guidelines to routinely prevent chronic vascular disease in primary care: the Preventive Evidence into Practice cluster randomised controlled trial. BMJ Open. 2015;5(12):e009397.

108. Hayashino Y, Suzuki H, Yamazaki K, Goto A, Izumi K, Noda M. A cluster randomized trial on the effect of a multifaceted intervention improved the technical quality of diabetes care by primary care physicians: The Japan Diabetes Outcome Intervention Trial-2 (J-DOIT2). Diabet Med. 2016;33(5):599-608.

109. Hayes R, Bratzler D, Armour B, Moore l. Comparison of an enhanced versus written feedback model on the management of Medicare inpatients with venous thrombosis. Jt Comm J Qual Improv. 2001;27(3):155-68.

110. Hayes RP, Baker DW, Luthi JC, Baggett RL, McClellan W, FitzGerald D. The effect of external feedback on the management of medicare inpatients with congestive heart failure. Am J Med Qual. 2002;17:225-35.

111. Hemkens LG, Saccilotto R, Reyes SL, Glinz D, Zumbrunn T, Grolimund O, et al. Personalized Prescription Feedback Using Routinely Collected Data to Reduce Antibiotic Use in Primary Care: A Randomized Clinical Trial. JAMA Intern Med. 2017;177(2):176-83.

112. Hemminiki E, Teperi J, Tuominen K. Need for and influence or feedback from the Finnish birth register to data providers. Int J Health Care Qual Assur. 1992;4(2):133-9.

113. Hendryx MS, Fieselmann JF, Bock MJ, Wakefield DS, Helms CM, Bentler SE. Outreach education to improve quality of rural icu care. Am J Respir Crit Care Med. 1998;158:418-23.

114. Herbert CP, Wright JM, Maclure M, Wakefield J, Dormuth C, Brett-MacLean P. Better Prescribing Project: a randomized controlled trial of the impact of case-based educational modules and personal prescribing feedback on prescribing for hypertension in primary care. Fam Pract. 2004;21:575-81.

115. Hermans MP, Elisaf M, Michel G, Muls E, Nobels F, Vandenberghe H, et al. Benchmarking is associated with improved quality of care in type 2 diabetes: the OPTIMISE randomized, controlled trial. Diabetes Care. 2013;36(11):3388-95.

116. Herrin J, Nicewander DA, Hollander PA, Couch CE, Winter FD, Haydar ZR. Effectiveness of diabetes resource nurse case management and physician profiling in a fee-for-service setting: a cluster randomized trial. Proceedings (Baylor University Medical Centre). 2006;19(2):95-102.

117. Hillman AL, Ripley K, Goldfarb N, Nuamah I, Weiner J, Lusk E. Physician financial incentives and feedback: Failure to increase cancer screening in medicaid managed care. Am J Public Health. 1998;88(11):1698-701.

118. Hocking JS, Temple-Smith M, Guy R, Donovan B, Braat S, Law M, et al. Population effectiveness of opportunistic chlamydia testing in primary care in Australia: a cluster-randomised controlled trial. Lancet. 2018;392(10156):1413-22.

119. Hogg W, Lemelin J, Graham ID, Grimshaw J, Martin C, Moore L, et al. Improving prevention in primary care: evaluating the effectiveness of outreach facilitation. Fam Pract. 2008;25(1):40-8.

120. Holm M. Intervention against long-term use if hypnotics/sedatives in general practice. Scand J Prim Health Care. 1990;8:113-7.

121. Horbar JD, Carpenter JH, Buzas J, Soll RF, Suresh G, Bracken MB, et al. Collaborative quality improvement to promote evidence based surfactant for preterm infants: a cluster randomised trial. BMJ. 2004;329(7473):1004.

122. Houston TK, Sadasivam RS, Allison JJ, Ash AS, Ray MN, English TM, et al. Evaluating the QUIT-PRIMO clinical practice ePortal to increase smoker engagement with online cessation interventions: a national hybrid type 2 implementation study. Implement Sci. 2015;10:154.

123. Huffman MD, Mohanan PP, Devarajan R, Baldridge AS, Kondal D, Zhao L, et al. Effect of a Quality Improvement Intervention on Clinical Outcomes in Patients in India With Acute Myocardial Infarction: The ACS QUIK Randomized Clinical Trial. JAMA. 2018;319(6):567-78.

124. Huis A, Schoonhoven L, Grol R, Donders R, Hulscher M, van Achterberg T. Impact of a team and leaders-directed strategy to improve nurses' adherence to hand hygiene guidelines: a cluster randomised trial. Int J Nurs Stud. 2013;50(4):464-74.

125. Hurlimann D, Limacher A, Schabel M, Zanetti G, Berger C, Muhlemann K, et al. Improvement of antibiotic prescription in outpatient care: a cluster-randomized intervention study using a sentinel surveillance network of physicians. J Antimicrob Chemother. 2015;70(2):602-8.

126. Hux JE, Melady MP, DeBoer D. Confidential prescriber feedback and education to improve antibiotic use in primary care: a controlled trial. CMAJ. 1999;161:388-92.

127. Ivers NM, Tu K, Young J, Francis JJ, Barnsley J, Shah BR, et al. Feedback GAP: pragmatic, cluster-randomized trial of goal setting and action plans to increase the effectiveness of audit and feedback interventions in primary care. Implement Sci. 2013;8:142.

128. Kabore C, Ridde V, Chaillet N, Yaya Bocoum F, Betran AP, Dumont A. DECIDE: a cluster-randomized controlled trial to reduce unnecessary caesarean deliveries in Burkina Faso. BMC Med. 2019;17(1):87.

129. Kahan NR, Kahan E, Waitman DA, Kitai E, Chintz DP. The tools of an evidence-based culture: implementing clinical-practice guidelines in an Israeli HMO. Acad Med. 2009;84(9):1217-25.

130. Kaminski MF, Anderson J, Valori R, Kraszewska E, Rupinski M, Pachlewski J, et al. Leadership training to improve adenoma detection rate in screening colonoscopy: a randomised trial. Gut. 2016;65(4):616-24.

131. Katz DA, Muehlenbruch DR, Brown RL, Fiore MC, Baker TB. Effectiveness of implementing the agency for healthcare research and quality smoking cessation clinical practice guideline: a randomized, controlled trial. J Natl Cancer Inst. 2004;96(8):594-603.

132. Kaufmann-Kolle P, Szecsenyi J, Broge B, Haefeli WE, Schneider A. [Does implementation of benchmarking in quality circles improve the quality of care of patients with asthma and reduce drug interaction?]. Z Evid Fortbild Qual Gesundh wesen. 2011;105(5):389-95.

133. Kennedy CC, Ioannidis G, Thabane L, Adachi JD, Marr S, Giangregorio LM, et al. Successful knowledge translation intervention in long-term care: final results from the vitamin D and osteoporosis study (ViDOS) pilot cluster randomized controlled trial. Trials. 2015;16:214.

134. Kerry S, Oakeshott P, Dundas D, Williams J. Influence of postal distribution of the royal college of radiologists guidelines, together with feedback on radiological referral rates, on x-ray referrals from general practice: a randomized controlled trial. Fam Pract. 2000;17(1):46-52.

135. Kiefe CI, Allison JJ, Williams OD, Person SD, Weaver MT, Weissman NW. Improving quality improvement using achievable benchmarks for physician feedback: a randomized controlled trial. JAMA. 2001;285(22):2871-9.

136. Kim CS, Kristopaitis RJ, Stone E, Pelter M, Sandhu M, Weingarten SR. Physician education and report cards: do they make the grade? Results from a randomized controlled trial. Am J Med. 1999;107:556-60.

137. Kinsinger LS, Harris R, Qaqish B, Strecher V, Kaluzny A. Using an office system intervention to increase breast cancer screening. J Gen Intern Med. 1998;13:507-14.

138. Kritchevsky SB, Braun BI, Bush AJ, Bozikis MR, Kusek L, Burke JP, et al. The effect of a quality improvement collaborative to improve antimicrobial prophylaxis in surgical patients: a randomized trial. Ann Intern Med. 2008;149(7):472-80, W89-93.

139. Lafata Jennifer E, Gunter Margaret J, Hsu J, Kaatz S, Krajenta R, Platt R, et al. Academic Detailing to Improve Laboratory Testing among Outpatient Medication Users. Med Care. 2007;45(10):966-72.

140. Lakshminarayan K, Borbas C, McLaughlin B, Morris NE, Vazquez G, Luepker RV. A cluster-randomized trial to improve stroke care in hospitals. Neurology. 2010;74:1634-42.

141. Lemelin J, Hogg W, Baskerville N. Evidence to action: a tailored multifaceted approach to changing family physician practice patterns and improving preventive care. CMAJ. 2001;164(6):757-63.

142. Lesuis N, van Vollenhoven RF, Akkermans RP, Verhoef LM, Hulscher ME, den Broeder AA. Rheumatologists' guideline adherence in rheumatoid arthritis: a randomised controlled study on electronic decision support, education and feedback. Clin Exp Rheumatol. 2018;36(1):21-8.

143. Levi CR, Attia JA, D'Este C, Ryan AE, Henskens F, Kerr E, et al. Cluster-Randomized Trial of Thrombolysis Implementation Support in Metropolitan and Regional Australian Stroke Centers: Lessons for Individual and Systems Behavior Change. J Am Heart Assoc. 2020;9(3):e012732.

144. Leviton LC, Goldenberg RL, Baker CS, Schwartz RM, Freda MC, Fish LJ, et al. Methods to encourage the use of antenatal corticosteroid therapy for fetal maturation: a randomized controlled trial. JAMA. 1999;281(1):46-52.

145. Lim WY, Hss AS, Ng LM, John Jasudass SR, Sararaks S, Vengadasalam P, et al. The impact of a prescription review and prescriber feedback system on prescribing practices in primary care clinics: a cluster randomised trial. BMC Fam Pract. 2018;19(1):120.

146. Linder JA, Schnipper JL, Tsurikova R, Yu DT, Volk LA, Melnikas AJ, et al. Electronic health record feedback to improve antibiotic prescribing for acute respiratory infections. Am J Manag Care. 2010;16(12 Suppl HIT):e311-9.

147. Lomas J, Enkin M, Anderson GM, Hannah WJ, Vayda E, Singer J. Opinion leaders vs audit and feedback to implement practice guidelines. Delivery after previous cesarean section. JAMA. 1991;265:2202-7.

148. Lopez-Picazo JJ, Ruiz JC, Sanchez JF, Ariza A, Aguilera B. A randomized trial of the effectiveness and efficiency of interventions to reduce potential drug interactions in primary care. Am J Med Qual. 2011;26(2):145-53.

149. Luitjes SHE, Hermens R, de Wit L, Heymans MW, van Tulder MW, Wouters M. An innovative implementation strategy to improve the use of Dutch guidelines on hypertensive disorders in pregnancy: A randomized controlled trial. Pregnancy Hypertens. 2018;14:131-8.

150. Lundborg CS, Wahlstrom R, Oke T, Tomson G, Diwan VK. Influencing prescribing for urinary tract infection and asthma in primary care in Sweden: a randomized controlled trial of an interactive educational intervention. J Clin Epidemiol. 1999;52(8):801-12.

151. Lundborg CS, Wahlstrom R, Oke T, Tomson G, Diwan VK. Influencing prescribing for urinary tract infection and asthma in primary care in Sweden: A randomized controlled trial of an interactive educational intervention. J Clin Epidemiol 1999;52(8):801-12.

152. Lynch EA, Cadilhac DA, Luker JA, Hillier SL. Education-only versus a multifaceted intervention for improving assessment of rehabilitation needs after stroke; a cluster randomised trial. Implement Sci. 2016;11(1):120.

153. Machline-Carrion MJ, Soares RM, Damiani LP, Campos VB, Sampaio B, Fonseca FH, et al. Effect of a Multifaceted Quality Improvement Intervention on the Prescription of Evidence-Based Treatment in Patients at High Cardiovascular Risk in Brazil: The BRIDGE Cardiovascular Prevention Cluster Randomized Clinical Trial. JAMA Cardiol. 2019;4(5):408-17.

154. Machline-Carrion MJ, Santucci EV, Damiani LP, Bahit MC, Malaga G, Pontes-Neto OM, et al. Effect of a Quality Improvement Intervention on Adherence to Therapies for Patients with Acute Ischemic Stroke and Transient Ischemic Attack: a Cluster Randomized Clinical Trial. JAMA Neurol. 2019.

155. Maddocks H, Stewart M, Thind A, Terry AL, Chevendra V, Marshall JN, et al. Feedback and training tool to improve provision of preventive care by physicians using EMRs: a randomised control trial. Inform Prim Care. 2011;19(3):147-53.

156. Manfredi C, Czaja R, Freels S, Trubitt M, Warnecke R, Lacey L. Prescribe for health. Improving cancer screening in physician practices serving low-income and minority populations. Arch Fam Med. 1998;7(4):329-37.

157. Martin AR, Wolf MA, Thibodeau LA, Dzau V, Braunwald E. A trial of two strategies to modify the test-ordering behavior of medical residents. N Engl J Med. 1980;303:1330-6.

158. Marton KI, Tul V, Sox HC, Jr. Modifying test-ordering behavior in the outpatient medical clinic. A controlled trial of two educational interventions. AMA Arch Intern Med. 1985;145:816-21.

159. Mayne SL, duRivage NE, Feemster KA, Localio AR, Grundmeier RW, Fiks AG. Effect of decision support on missed opportunities for human papillomavirus vaccination. Am J Prev Med. 2014;47(6):734-44.

160. McCartney P, Macdowall W, Thorogood M. A randomised controlled trial of feedback to general, practitioners of their prophylactic aspirin prescribing. BMJ. 1997;315:35-6.

161. McClellan WM, Millman L, Presley R, Couzins J, Flanders WD. Improved diabetes care by primary care physicians: results of a group-randomized evaluation of the Medicare Health Care Quality Improvement Program (HCQIP). J Clin Epidemiol. 2003;56:1210-7.

162. McClellan WM, Hodgin E, Pastan S, McAdams L, Soucie M. A randomized evaluation of two health care quality improvement program (HCQIP) interventions to improve the adequacy of hemodialysis care of ESRD patients: feedback alone versus intensive intervention. J Am Soc Nephrol. 2004;15:754-60.

163. McCluskey A, Ada L, Kelly PJ, Middleton S, Goodall S, Grimshaw JM, et al. A behavior change program to increase outings delivered during therapy to stroke survivors by community rehabilitation teams: The Out-and-About trial. Int J. 2016;11(4):425-37.

164. McCollell TS, Cushing AH, Healy JL, McIlvenna PA, Skipper BJ. Physician behavior modification using claims data: tetracycline for upper respiratory infection. West J Med. 1982;137(5):448-50.

165. Meeker D, Linder JA, Fox CR, Friedberg MW, Persell SD, Goldstein NJ, et al. Effect of Behavioral Interventions on Inappropriate Antibiotic Prescribing Among Primary Care Practices: A Randomized Clinical Trial. JAMA. 2016;315(6):562-70.

166. Mertens JR, Chi FW, Weisner CM, Satre DD, Ross TB, Allen S, et al. Physician versus non-physician delivery of alcohol screening, brief intervention and referral to treatment in adult primary care: the ADVISe cluster randomized controlled implementation trial. Addict Sci Clin Pract. 2015;10:26.

167. Metlay JP, Camargo C, MacKenzie T, McCulloch C, Maselli J, Levin SK, et al. Cluster-randomized trial to improve antibiotic use for adults with acute respiratory infections treated in emergency departments. Ann Emerg Med. 2007;50(3):221-30.

168. Mitchell E, Sullivan F, Grimshaw JM, Donnan PT, Watt G. Improving management of hypertension in general practice: a randomised controlled trial of feedback derived from electronic patient data. Br J Gen Pract. 2005;55(511):94-101.

169. Moher M, Yudkin P, Wright L, Turner R. Cluster randomised controlled trial to compare three methods of promoting secondary prevention of coronary hearth disease in primary care. BMJ. 2001;322(7298):1338.

170. Mold JW, Aspy CA, Nagykaldi Z, Oklahoma Physicians Resource/Research N. Implementation of evidence-based preventive services delivery processes in primary care: an Oklahoma Physicians Resource/Research Network (OKPRN) study. J Am Board Fam Med. 2008;21(4):334-44.

171. Mold JW, Fox C, Wisniewski A, Lipman PD, Krauss MR, Harris DR, et al. Implementing asthma guidelines using practice facilitation and local learning collaboratives: a randomized controlled trial. Ann Fam Med. 2014;12(3):233-40.

172. Morrison LJ, Brooks SC, Dainty KN, Dorian P, Needham DM, Ferguson ND, et al. Improving use of targeted temperature management after out-of-hospital cardiac arrest: a stepped wedge cluster randomized controlled trial. Crit Care Med. 2015;43(5):954-64.

173. Myers RE, Turner B, Weinberg D, Hyslop T, Hauck WW, Brigham T, et al. Impact of a physician-oriented intervention on follow-up in colorectal cancer screening. Prev Med. 2004;38(4):375-81.

174. Nace DA, Hanlon JT, Crnich CJ, Drinka PJ, Schweon SJ, Anderson G, et al. A Multifaceted Antimicrobial Stewardship Program for the Treatment of Uncomplicated Cystitis in Nursing Home Residents. JAMA Intern Med. 2020.

175. Naughton C, Feely J, Bennett K. A clustered randomized trial of the effects of feedback using academic detailing compared to postal bulletin on prescribing of preventative cardiovascular therapy. Fam Pract. 2007;24(5):475-80.

176. Naughton C, Feely J, Bennett K. A RCT evaluating the effectiveness and cost-effectiveness of academic detailing versus postal prescribing feedback in changing GP antibiotic prescribing. J Eval Clin Pract. 2009;15(5):807-12.

177. Navarro HJ, Shakeshaft A, Doran CM, Petrie DJ. The cost-effectiveness of tailored, postal feedback on general practitioners' prescribing of pharmacotherapies for alcohol dependence. Drug Alcohol Depend. 2012;124(3):207-15.

178. Navathe AS, Volpp KG, Bond AM, Linn KA, Caldarella KL, Troxel AB, et al. Assessing The Effectiveness Of Peer Comparisons As A Way To Improve Health Care Quality. Health Aff. 2020;39(5):852-61.

179. Nejad AS, Noori MR, Haghdoost AA, Bahaadinbeigy K, Abu-Hanna A, Eslami S. The effect of registry-based performance feedback via short text messages and traditional postal letters on prescribing parenteral steroids by general practitioners--A randomized controlled trial. Int J Med Inf. 2016;87:36-43.

180. Nilsson G, Hjemdal P, Hassler A, Vitols S, Wallen NH, Krakau I. Feedback on prescribing rate combined with problem-oriented pharmacotherapy education as a model to improve prescribing among general practitioners. Eur J Clin Pharmacol. 2001;56(11):843-8.

181. Nilsson G, Hjemdal P, Hassler A, Vitols S, Wallen NH, Krakau I. Feedback on prescribing rate combined with problem-oriented pharmacotherapy education as a model to improve prescribing among general practitioners. Eur J Clin Pharmacol 2001;56(11):843-8.

182. O´Connell DL, Henry D, Tomlins R. Randomised controlled trial of effect of feedback on general practitioners prescribing in Australia. BMJ. 1999;318:507-11.

183. O'Connor PJ, Sperl-Hillen J, Johnson PE, Rush WA, Crain AL. Customized feedback to patients and providers failed to improve safety or quality of diabetes care: a randomized trial. Diabetes Care. 2009;32(7):1158-63.

184. Ornstein S, Jenkins RG, Nietert PJ, Feifer C, Roylance LF, Nemeth L. A multimethod quality improvement intervention to improve preventive cardiovascular care: a cluster randomized trial. Ann Intern Med. 2004;141(7):523-32.

185. Ornstein S, Nemeth LS, Jenkins RG, Nietert PJ. Colorectal cancer screening in primary care: translating research into practice. Med Care. 2010;48(10):900-6.

186. Overbeek LI, Hermens RP, van Krieken JH, Adang EM, Casparie M, Nagengast FM, et al. Electronic reminders for pathologists promote recognition of patients at risk for Lynch syndrome: cluster-randomised controlled trial. Virchows Arch. 2010;456(6):653-9.

187. Palmer RH, Louis TA, Hsu LN, Peterson HF, Rothrock JK, Strain R, et al. A randomized controlled trial of quality assurance in sixteen ambulatory care practices. Med Care. 1985;23:751-70.

188. Palmer RH, Louis TA, Hsu LN, Peterson HF, Rothrock JK, Strain R, et al. A randomized controlled trial of quality assurance in sixteen ambulatory care practices. Med Care 1985;23:751-70.

189. Papadakis S, Cole AG, Reid RD, Assi R, Gharib M, Tulloch HE, et al. From Good to Great: The Role of Performance Coaching in Enhancing Tobacco-Dependence Treatment Rates. Ann Fam Med. 2018;16(6):498-506.

190. Pape GA, Hunt JS, Butler KL, Siemienczuk J, LeBlanc BH, Gillanders W, et al. Team-based care approach to cholesterol management in diabetes mellitus: two-year cluster randomized controlled trial. Arch Intern Med. 2011;171(16):1480-6.

191. Patel M, Kurtzman GW, Kannan S, Small D, Morris A, Honeywell S, et al. Effect of an automated patient dashboard using active choice and peer comparison performance feedback to physicians on statin prescribing: the pre-scribe randomized clinical trial. J Gen Intern Med. 2018;33(2):174‐5.

192. Patel S, Rajkomar A, Harrison JD, Prasad PA, Valencia V, Ranji SR, et al. Next-generation audit and feedback for inpatient quality improvement using electronic health record data: a cluster randomised controlled trial. BMJ Qual Saf. 2018;27(9):691-9.

193. Pavese P, Maillet M, Vitrat-Hincky V, Recule C, Vittoz JP, Guyomard A, et al. Evaluation of an intervention to improve blood culture practices: a cluster randomised trial. Eur J Clin Microbiol Infect Dis. 2014;33(12):2207-13.

194. Peiris D, Usherwood T, Panaretto K, Harris M, Hunt J, Redfern J, et al. Effect of a computer-guided, quality improvement program for cardiovascular disease risk management in primary health care: the treatment of cardiovascular risk using electronic decision support cluster-randomized trial. Circ Cardiovasc Qual Outcomes. 2015;8(1):87-95.

195. Persell SD, Liss DT, Walunas TL, Ciolino JD, Ahmad FS, Brown T, et al. Effects of 2 Forms of Practice Facilitation on Cardiovascular Prevention in Primary Care: A Practice-randomized, Comparative Effectiveness Trial. Med Care. 2020;58(4):344-51.

196. Peters-Klimm F, Muller-Tasch T, Remppis A, Szecsenyi J, Schellberg D. Improved guideline adherence to pharmacotherapy of chronic systolic heart failure in general practice--results from a cluster-randomized controlled trial of implementation of a clinical practice guideline. J Eval Clin Pract. 2008;14(5):823-9.

197. Petersen LA, Simpson K, Pietz K, Urech TH, Hysong SJ, Profit J, et al. Effects of individual physician-level and practice-level financial incentives on hypertension care: a randomized trial. JAMA. 2013;310(10):1042-50.

198. Pettersson E, Vernby A, Molstad S, Lundborg CS. Can a multifaceted educational intervention targeting both nurses and physicians change the prescribing of antibiotics to nursing home residents? A cluster randomized controlled trial. J Antimicrob Chemother. 2011;66(11):2659-66.

199. Pimlott NJG, Hux JE, Wilson LM, Kahan M, Li C, Rosser WW. Educating physicians to reduce benzodiazepine use by elderly patients: a randomized controlled trial. CMAJ. 2003;168:835-9.

200. Price-Haywood EG, Harden-Barrios J, Cooper LA. Comparative effectiveness of audit-feedback versus additional physician communication training to improve cancer screening for patients with limited health literacy. J Gen Intern Med. 2014;29(8):1113-21.

201. Quanbeck A, Brown RT, Zgierska AE, Jacobson N, Robinson JM, Johnson RA, et al. A randomized matched-pairs study of feasibility, acceptability, and effectiveness of systems consultation: a novel implementation strategy for adopting clinical guidelines for Opioid prescribing in primary care. Implement Sci. 2018;13(1):21.

202. Quinley JC, Shih A. Improving physician coverage of pneumcoccal vaccine: a randomized trial of telephone intervention. J Community Health. 2004;29:103-15.

203. Raasch BA, Hays R, Buettner PG. An educational intervention to improve diagnosis and management of suspicious skin lesions. J Contin Educ Health Prof. 2000;20:39-51.

204. Raja AS, Ip IK, Dunne RM, Schuur JD, Mills AM, Khorasani R. Effects of Performance Feedback Reports on Adherence to Evidence-Based Guidelines in Use of CT for Evaluation of Pulmonary Embolism in the Emergency Department: A Randomized Trial. AJR Am J Roentgenol. 2015;205(5):936-40.

205. Ralph AP, de Dassel JL, Kirby A, Read C, Mitchell AG, Maguire GP, et al. Improving Delivery of Secondary Prophylaxis for Rheumatic Heart Disease in a High-Burden Setting: Outcome of a Stepped-Wedge, Community, Randomized Trial. J Am Heart Assoc. 2018;7(14).

206. Rantz MJ, Popejoy L, Petroski GF, Madsen RW, Mehr DR, Zwygart-Stauffacher M. Randomized clinical trial of quality improvement intervention in nursing homes. Gerontologist. 2001;41(4):525-38.

207. Rask K, Kohler SA, Wells KJ, Williams JA, Diamond CC. Performance improvement interventions to improve delivery of screening services in diabetes care. J Clin Outcomes Manag. 2001;8:23-9.

208. Robling MR, Houston HL, Kinnersley P, Hourihan MD, Cohen DR, Hale J. General practitioners' use of magnetic resonance imaging: an open randomized trial comparing telephone and written requests and an open randomized controlled trial of different methods of local guideline dissemination. Clin Radiol. 2002;57(5):402-7.

209. Rodriguez V, Giuffre C, Villa S, Almada G, Prasopa-Plaizier N, Gogna M, et al. A multimodal intervention to improve hand hygiene in ICUs in Buenos Aires, Argentina: a stepped wedge trial. Int J Qual Health Care. 2015;27(5):405-11.

210. Roos-Blom MJ, Gude WT, de Jonge E, Spijkstra JJ, van der Veer SN, Peek N, et al. Impact of audit and feedback with action implementation toolbox on improving ICU pain management: cluster-randomised controlled trial. BMJ Qual Saf. 2019;28(12):1007-15.

211. Ruangkanchanasetr S. Laboratory investigation utilization in pediatric out-patient department ramathibodi hospital. J Med Assoc Thai. 1993;76:194-9.

212. Ruangkanchanasetr S. Laboratory investigation utilization in pediatric out-patient department ramathibodi hospital. J Med Assoc Thai 1993;76:194-9.

213. Ruangkanchanasetr SLiuipo-pdrh. Laboratory investigation utilization in pediatric out-patient department ramathibodi hospital. J Med Assoc Thai 1993;76:194-9.

214. Rubin GL, Schofield WN, Dean MG, Shakeshaft AP. Appropriateness of red blood cell transfusions in major urban hospitals and effectiveness of an intervention. Med J Aust. 2001;175:354-8.

215. Rust CT, Sisk FA, Kuo AR, Smith J, Miller R, Sullivan KM. Impact of resident feedback on immunization outcomes. Arch Pediatr Adolesc Med. 1999;153:1165-9.

216. Ryan AM, McCullough CM, Shih SC, Wang JJ, Ryan MS, Casalino LP. The intended and unintended consequences of quality improvement interventions for small practices in a community-based electronic health record implementation project. Med Care. 2014;52(9):826-32.

217. Ryskina K, Jessica Dine C, Gitelman Y, Leri D, Patel M, Kurtzman G, et al. Effect of Social Comparison Feedback on Laboratory Test Ordering for Hospitalized Patients: A Randomized Controlled Trial. J Gen Intern Med. 2018;33(10):1639-45.

218. Sauaia A, Ralston D, Schluter WW, Marciniak TA, Havranek EP, Dunn TR. Influencing care in acute myocardial infarction: a randomized trial comparing 2 types of intervention. Am J Med Qual. 2000;15:197-206.

219. Scales DC, Dainty K, Hales B, Pinto R, Fowler RA, Adhikari NK, et al. A multifaceted intervention for quality improvement in a network of intensive care units: a cluster randomized trial. JAMA. 2011;305(4):363-72.

220. Scales DC, Golan E, Pinto R, Brooks SC, Chapman M, Dale CM, et al. Improving Appropriate Neurologic Prognostication after Cardiac Arrest. A Stepped Wedge Cluster Randomized Controlled Trial. Am J Respir Crit Care Med. 2016;194(9):1083-91.

221. Schectman JM, Kanwal NK, Schroth WS, Elinsky EG. The effect of an education and feedback intervention on group-model and network-model health maintenance organization physician prescribing behavior. Med Care. 1995;33:139-44.

222. Schectman JM, Schroth WS, Verme D, Voss JD. Randomized controlled trial of education and feedback for implementation of guidelines for acute low back pain. J Gen Intern Med. 2003;18:773-80.

223. Schmidt-Mende K, Andersen M, Wettermark B, Hasselstrom J. Educational intervention on medication reviews aiming to reduce acute healthcare consumption in elderly patients with potentially inappropriate medicines-A pragmatic open-label cluster-randomized controlled trial in primary care. Pharmacoepidemiol Drug Saf. 2017;26(11):1347-56.

224. Schneider A, Wensing M, Biessecker K, Quinzler R, Kaufmann-Kolle P, Szecsenyi J. Impact of quality circles for improvement of asthma care: results of a randomized controlled trial. J Eval Clin Pract. 2008;14(2):185-90.

225. Scholes D, Grothaus L, McClure J, Reid R, Fishman P, Sisk C. A randomized trial of strategies to increase chlamydia screening in young women. Prev Med. 2006;43(4):343-50.

226. Shen X, Lu M, Feng R, Cheng J, Chai J, Xie M, et al. Web-Based Just-in-Time Information and Feedback on Antibiotic Use for Village Doctors in Rural Anhui, China: Randomized Controlled Trial. J Med Internet Res. 2018;20(2):e53.

227. Sinclair C, Frankel M. The effect of quality assurance activities on the quality of mental health services. QRB Qual Rev Bull. 1982;8(7):7-15.

228. Siriwardena AN, Rashid A, Johnson MRD, Dewey ME. Cluster randomised controlled trial of an educational outreach visit to improve influenza and pneumococcal immunisation rates in primary care. Br J Gen Pract. 2002;52:735-40.

229. Smith DK, Shaw RW, Slack J, Marteau TM. Training obstetricians and midwives to present screening tests evaluation of two brief interventions. Prenat Diagn. 1995;15:317-24.

230. Smith-Bindman R, Chu P, Wang Y, Chung R, Lopez-Solano N, Einstein AJ, et al. Comparison of the Effectiveness of Single-Component and Multicomponent Interventions for Reducing Radiation Doses in Patients Undergoing Computed Tomography: A Randomized Clinical Trial. JAMA Intern Med. 2020.

231. Socolar RR, Raines B, Chen-Mok M, Runyan DK, Green C, Paterno S. Intervention to improve physician documentation and knowledge of child sexual abuse: a randomized, controlled trial. Pediatrics. 1998;101(5):817-24.

232. Soleymani F, Rashidian A, Hosseini M, Dinarvand R, Kebriaeezade A, Abdollahi M. Effectiveness of audit and feedback in addressing over prescribing of antibiotics and injectable medicines in a middle-income country: an RCT. DARU. 2019.

233. Solomon DH, Katz JN, La Tourette AM, Coblyn JS. Multifaceted intervention to improve rheumatologists' management of glucocorticoid-induced osteoporosis: a randomized controlled trial. Semin Arthritis Rheum. 2004;51(3):383-7.

234. Soumerai SB, McLaughlin TJ, Gurwitz JH, Guadagnoli E, Hauptman PJ, Borbas C. Effect of local medical opinion leaders on quality of care for acute myocardial infarction: a randomized controlled trial. JAMA. 1998;279(17):1358-63.

235. Spoorenberg V, Hulscher ME, Geskus RB, de Reijke TM, Opmeer BC, Prins JM, et al. A Cluster-Randomized Trial of Two Strategies to Improve Antibiotic Use for Patients with a Complicated Urinary Tract Infection. PLoS ONE. 2015;10(12):e0142672.

236. Stewardson AJ, Sax H, Gayet-Ageron A, Touveneau S, Longtin Y, Zingg W, et al. Enhanced performance feedback and patient participation to improve hand hygiene compliance of health-care workers in the setting of established multimodal promotion: a single-centre, cluster randomised controlled trial. Lancet Infect Dis. 2016;16(12):1345-55.

237. Søndergaard J, Adersen M, Vach K, Kragstrup J, Maclure M, Gram LF. Detailed postal feedback about prescribing to asthma patients combined with a guideline statement showed no impact: a randomised controlled trial. Eur J Clin Pharmacol. 2002;58:127-32.

238. Søndergaard J, Andersen M, Støvring H, Kragstrup J. Mailed prescribed feedback in addition to a clinical guideline has no impact: a randomised, controlled trial. Scand J Prim Health Care. 2003;21:47-51.

239. Søndergaard J, Hansen DG, Aarslev P, Munck AP. A multifaceted intervention according to the Audit Project Odense method improved secondary prevention of ischemic heart disease: a randomised controlled trial. Fam Pract. 2006;23(2):198-202.

240. Tadrous M, Fung K, Desveaux L, Gomes T, Taljaard M, Ivers N. Appropriate prescribing in nursing homes demonstration project: a pragmatic, cluster-randomized trial. Pharmacoepidemiol Drug Saf. 2019;28:398‐.

241. Thomas RE, Croal BL, Ramsay C, Eccles M, Grimshaw J. Effect of enhanced feedback and brief educational reminder messages on laboratory test requesting in primary care: a cluster randomised trial. Lancet. 2006;367:1990-6.

242. Thomas KG, Thomas MR, Stroebel RJ, McDonald FS, Hanson GJ, Naessens JM. Use of a registry-generated audit, feedback, and patient reminder intervention in an internal medicine resident clinic--a randomized trial. J Gen Intern Med. 2007;22:1740-4.

243. Tianviwat S, Hintao J, Chongsuvivatwong V, Thitasomakul S. A randomized controlled trial of cluster audit and feedback on the quality of dental sealant for rural schoolchildren. Community Dent Health. 2016;33(1):27-32.

244. Tierney WM, Hui SL, McDonald CJ. Delayed feedback of physician performance versus immediate reminders to perform preventive care. Effects on physician compliance. Med Care. 1986;24(8):659-66.

245. Tierney WM, Hui SL, McDonald CJ. Delayed feedback of physician performance versus immediate reminders to perform preventive care. Effects on physician compliance. Med Care 1986;24(8):659-66.

246. Trent SA, Havranek EP, Ginde AA, Haukoos JS. Effect of Audit and Feedback on Physician Adherence to Clinical Practice Guidelines for Pneumonia and Sepsis. Am J Med Qual. 2019;34(3):217-25.

247. Trietsch J, van Steenkiste B, Grol R, Winkens B, Ulenkate H, Metsemakers J, et al. Effect of audit and feedback with peer review on general practitioners' prescribing and test ordering performance: a cluster-randomized controlled trial. BMC Fam Pract. 2017;18(1):53.

248. Trietsch J, van Steenkiste B, Grol R, Winkens B, Ulenkate H, Metsemakers J, et al. Effect of audit and feedback with peer review on general practitioners' prescribing and test ordering performance: a cluster-randomized controlled trial. BMC Fam Pract 2017;18(1):53.

249. Tu JV, Donovan LR, Lee DS, Wang JT, Austin PC, Alter DA. Effectiveness of public report cards for improving the quality of cardiac care: the EFFECT study: a randomized trial. JAMA. 2009;302(21):2330-7.

250. van Bruggen R, Gorter KJ, Stolk RP, Verhoeven RP, Rutten GE. Implementation of locally adapted guidelines on type 2 diabetes. Fam Pract. 2008;25(6):430-7.

251. van der Velden AW, Kuyvenhoven MM, Verheij TJ. Improving antibiotic prescribing quality by an intervention embedded in the primary care practice accreditation: the ARTI4 randomized trial. J Antimicrob Chemother. 2016;71(1):257-63.

252. Van der Weijden T, Grol RP, Knottinerus JA. Feasibility of a national cholestrol guideline in daily practice. A randomized controlled trial in 20 general practices. Int J Qual Health Care. 1999;11(2):131-7.

253. Vellinga A, Galvin S, Duane S, Callan A, Bennett K, Cormican M, et al. Intervention to improve the quality of antimicrobial prescribing for urinary tract infection: A cluster randomized trial. CMAJ. 2016;188(2):108-15.

254. Verstappen WH, van der Weijden T, Sijbrandij J, Smeele I, Hermsen J, Grimshaw J, et al. Effect of a practice-based strategy on test ordering performance of primary care physicians. JAMA. 2003;289(18):2407-12.

255. Verstappen WH, van der Weijden T, Sijbrandij J, Smeele I, Hermsen J, Grimshaw J, et al. Effect of a practice-based strategy on test ordering performance of primary care physicians. JAMA. 2003;289:2407-12.

256. Verstappen WHJM, Van der Weijden T, Dubois WI, Smeele I, Hermsen J, Tan FES. Improving test ordering in primary care: the added value of a small-group quality improvement strategy compared with classic feedback only. Ann Fam Med. 2004;2:569-75.

257. Vervloet M, Meulepas MA, Cals JW, Eimers M, van der Hoek LS, van Dijk L. Reducing antibiotic prescriptions for respiratory tract infections in family practice: results of a cluster randomized controlled trial evaluating a multifaceted peer-group-based intervention. NPJ Prim Care Respir Med. 2016;26:15083.

258. Vingerhoets B, Wensing M, Grol R. Feedback of patients' evaluations of general practice care: a randomised trial. Qual Health Care. 2001;10:224-8.

259. von Lengerke T, Ebadi E, Schock B, Krauth C, Lange K, Stahmeyer JT, et al. Impact of psychologically tailored hand hygiene interventions on nosocomial infections with multidrug-resistant organisms: results of the cluster-randomized controlled trial PSYGIENE. Antimicrob. 2019;8:56.

260. Wadland WC, Holtrop JS, Weismantel D, Pathak PK, Fadel H, Powell J. Practice-based referrals to a tobacco cessation quit line: assessing the impact of comparative feedback vs general reminders. Ann Fam Med. 2007;5(2):135-42.

261. Wahlström R, Kounnavong S, Sisounthone B, Phanyanouvong A, Southammavong T, Eriksson B. Effectiveness of feedback for improving case management of malaria, diarrhoea and pneumonia - a randomized controlled trial at provincial hospitals in Lao PDR. Trop Med Int Health. 2003;8:901-9.

262. Wald HL, Bandle B, Richard AA, Min SJ, Capezuti E. A Trial of electronic surveillance feedback for quality improvement at Nurses Improving Care for Healthsystem Elders (NICHE) hospitals. Am J Infect Control. 2014;42(10 Suppl):S250-6.

263. Walsh M, Laptook A, Kazzi SN, Engle WA, Yao Q, Rasmussen M, et al. A cluster-randomized trial of benchmarking and multimodal quality improvement to improve rates of survival free of bronchopulmonary dysplasia for infants with birth weights of less than 1250 grams. Pediatrics. 2007;119(5):876-90.

264. Wang Y, Li Z, Zhao X, Wang C, Wang X, Wang D, et al. Effect of a Multifaceted Quality Improvement Intervention on Hospital Personnel Adherence to Performance Measures in Patients With Acute Ischemic Stroke in China: A Randomized Clinical Trial. JAMA. 2018;320(3):245-54.

265. Ward A, Kamien M, Mansfield F, Fatovich B. Educational feedback in management of diabetes in general practice. Educ Gen Pract. 1996;7:142-50.

266. Wathne JS, Kleppe LKS, Harthug S, Blix HS, Nilsen RM, Charani E, et al. The effect of antibiotic stewardship interventions with stakeholder involvement in hospital settings: a multicentre, cluster randomized controlled intervention study. Antimicrob. 2018;7:109.

267. Wattal C, Goel N, Khanna S, Byotra SP, Laxminarayan R, Easton A. Impact of informational feedback to clinicians on antibiotic-prescribing rates in a tertiary care hospital in Delhi. Indian J. 2015;33(2):255-9.

268. Wei X, Zhang Z, Walley JD, Hicks JP, Zeng J, Deng S, et al. Effect of a training and educational intervention for physicians and caregivers on antibiotic prescribing for upper respiratory tract infections in children at primary care facilities in rural China: a cluster-randomised controlled trial. Lancet Glob Health.5(12):e1258-67.

269. Wells KB, Sherbourne C, Schoenbaum M, Duan N, Meredith L, Unutzer J, et al. Impact of disseminating quality improvement programs for depression in managed primary care: a randomized controlled trial. JAMA. 2000;283(2):212-20.

270. Welschen I, Kuyvenhoven MM, Hoes AW, Verheij TJ. Effectiveness of a multiple intervention to reduce antibiotic prescribing for respiratory tract symptoms in primary care: randomised controlled trial. BMJ. 2004;329(7463):431.

271. Whidden C, Kayentao K, Liu JX, Lee S, Keita Y, Diakite D, et al. Improving Community Health Worker performance by using a personalised feedback dashboard for supervision: a randomised controlled trial. J Glob Health. 2018;8(2):020418.

272. Williams L, Daggett V, Slaven JE, Yu Z, Sager D, Myers J, et al. A cluster-randomised quality improvement study to improve two inpatient stroke quality indicators. BMJ Qual Saf. 2016;25(4):257-64.

273. Willis TA, Collinson M, Glidewell L, Farrin AJ, Holland M, Meads D, et al. An adaptable implementation package targeting evidence-based indicators in primary care: A pragmatic cluster-randomised evaluation. PLoS Med. 2020;17(2):e1003045.

274. Willis TA, Collinson M, Glidewell L, Farrin AJ, Holland M, Meads D, et al. An adaptable implementation package targeting evidence-based indicators in primary care: A pragmatic cluster-randomised evaluation. PLoS Med. 2020;17(2):e1003045.

275. Willis TA, Collinson M, Glidewell L, Farrin AJ, Holland M, Meads D, et al. An adaptable implementation package targeting evidence-based indicators in primary care: A pragmatic cluster-randomised evaluation. PLoS Med. 2020;17(2):e1003045.

276. Willis TA, Collinson M, Glidewell L, Farrin AJ, Holland M, Meads D, et al. An adaptable implementation package targeting evidence-based indicators in primary care: A pragmatic cluster-randomised evaluation. PLoS Med. 2020;17(2):e1003045.

277. Winickoff RN, Coltin KL, Morgan MM, Buxbaum RC, Barnett GO. Improving physician performance through peer comparison feedback. Med Care. 1984;22:527-34.

278. Winkens RA, Pop P, Bugter-Maessen AM, Grol RP, Kester AD, Beusmans GH, et al. Randomised controlled trial of routine individual feedback to improve rationality and reduce numbers of test requests. Lancet. 1995;345:498-502.

279. Winkens RA, Pop P, Bugter-Maessen AM, Grol RP, Kester AD, Beusmans GH, et al. Randomised controlled trial of routine individual feedback to improve rationality and reduce numbers of test requests. Lancet. 1995;345:498-502.

280. Winslade N, Eguale T, Tamblyn R. Optimising the changing role of the community pharmacist: a randomised trial of the impact of audit and feedback. BMJ Open. 2016;6(5):e010865.

281. Winslade N, Eguale T, Tamblyn R. Optimising the changing role of the community pharmacist: a randomised trial of the impact of audit and feedback. BMJ Open. 2016;6(5):e010865.

282. Wones RG. Failure of low-cost audits with feedback to reduce laboratory test utilization. Med Care. 1987;25:78-82.

283. Wu Y, Li S, Patel A, Li X, Du X, Wu T, et al. Effect of a Quality of Care Improvement Initiative in Patients With Acute Coronary Syndrome in Resource-Constrained Hospitals in China: A Randomized Clinical Trial. JAMA Cardiol. 2019;4(5):418-27.

284. Yano Elizabeth M. The role of organizational research in implementing evidence-based practice: QUERI Series. Implement Sci. 2008;3(1):29.

285. Zafar HM, Ip IK, Mills AM, Raja AS, Langlotz CP, Khorasani R. Effect of clinical decision support-generated report cards versus real-time alerts on primary care provider guideline adherence for low back pain outpatient lumbar spine MRI orders. AJR Am J Roentgenol. 2019;212(2):386-94.

286. Ziemer DC, Doyle JP, Barnes CS, Branch WTJ, Cook CB, El Kebbi IM. An intervention to overcome clinical inertia and improve diabetes mellitus control in a primary care setting: Improving Primary Care of African Americans with Diabetes (IPCAAD) 8. AMA Arch Intern Med. 2006;166:507-13.

287. Zimmerman RK, Nowalk MP, Lin CJ, Hannibal K, Moehling KK, Huang HH, et al. Cluster randomized trial of a toolkit and early vaccine delivery to improve childhood influenza vaccination rates in primary care. Vaccine. 2014;32(29):3656-63.

288. Zwar N, Wolk J, Gordon J, Fisher RS, Kehoe L. Influencing antibiotic prescribing in general practice: a trial of prescriber feedback and management guidelines. Fam Pract. 1999;16(5):495-500.

* Billue 2012 was a co-publication, data therefore merged with Estrada 2011
