# Supplementary material for: Identifying behaviour change techniques in 287 randomized controlled trials of audit and feedback interventions targeting practice change among healthcare professionals
Source: Implement Sci. 2023 Nov 21;18:63. doi: 10.1186/s13012-023-01318-8 (PMC10664600; doi:10.1186/s13012-023-01318-8)
Supplement: Supplementary file 2 — Additional file 2: Appendix 2. Coding framework for behaviour change techniques, operationalized for audit and feedback interventions. [file 13012_2023_1318_MOESM2_ESM.docx]

***Appendix 2.* Coding framework for behaviour change techniques, operationalized for audit and feedback interventions.**

| **Behaviour Change Technique** | **Original description** | **A&F specific heuristics** | **Examples of what to code** |
| --- | --- | --- | --- |
| **1.1 Goal setting (behaviour)** | Set or agree on a goal defined in terms of the behaviour to be achieved.   ***Note:*** *only code goal-setting if there is sufficient evidence that goal set as part of intervention; if goal unspecified or a behavioural outcome, code 1.3, Goal setting (outcome); if the goal defines a specific context, frequency, duration or intensity for the behaviour, also code 1.4, Action planning.* | Goal setting can be implied when clear behavioural targets, frequently based on clinical practice guidelines, are established and communicated with HCPs prior to the receipt of feedback. Goal setting (behaviour) can involve both choosing a desired performance level (e.g., using data from baseline assessments) to be achieved or setting objectives/strategies that are not attached to a specific performance level. Common examples of behavioural goals in the healthcare context relate to HCP referral and/or testing practices. Where it isn’t clear if goals relate to ‘behaviour’ or ‘outcomes’, check the outcomes reported in the Results section; if still unclear, code both (see 1.3). When set goals/targets (behaviour) are presented alongside feedback, consider coding 1.6 Discrepancy between current behaviour and goal  ***Key words***: targets, metrics, standards, thresholds  Heuristics informed by Presseau et al. (2015) | "Radiologists were able to insert their goals for changes they would like to make in their clinical practice, especially regarding recall rates, into a text field at the end of each module" (Carney, 2012)   "Finally participants set their own, internal targets guided by the information presented to increase target" (Roos-Blom, 2019) |
| **1.2 Problem solving** | Analyze, or prompt the person to analyze, factors influencing the behaviour and generate or select strategies that include overcoming barriers and/or increasing facilitators (includes ‘Relapse Prevention’ and ‘Coping Planning’).   Note: barrier identification without solutions is not sufficient. If the BCT does not include analyzing the behavioural problem, consider 11.2, 12.1, 12.2 or 12.3. | Problem solving with HCPs can involve gap analyses, fishbone analyses, or similar. Ideally it involves generating barriers and solutions towards achieving an audit standard; however, the generation of solutions can be inferred if a discussion of potential barriers/obstacles has taken place with the HCP/s. Also code problem solving when there is evidence of **coping planning** - planning that is designed to address anticipated moment-to-moment, potentially recurring situations that have the potential to side-track performing a behaviour.  Do not code problem solving conducted as part of intervention development (e.g., needs analysis).  ***Key words***: solutions, barriers, obstacles | "The groups were encouraged to share experiences and to work together to identify solutions" (Cundill, 2015)  "Meetings with local champions allowed us to discuss barriers to quality end-of-life care in their units and strategize about ways to address those barriers” (Curtis, 2011)  "The program encouraged discussion of current practices, reviewed the case for change, elicited barriers, and proposed solutions to barriers" (Lafata, 2007) |
| **1.3 Goal setting (outcome)** | Set or agree on a goal defined in terms of a positive outcome of wanted behaviour  ***Note:*** *only code guidelines if set as a goal in an intervention context; if goal is a behaviour, code 1.1, Goal setting (behaviour); if goal unspecified code 1.3,* Goal setting (outcome) | Goal setting can be implied when clear outcome targets, frequently based on clinical practice guidelines, are established and communicated with HCPs prior to the receipt of feedback. The outcome is often a clinical outcome, at either the HCP or patient level, but could also be a financial outcome. Common examples of outcome goals in the healthcare context include decreased incidence of disease, decreased hospitalisation rates, and reduced healthcare costs. Where it isn’t clear if goals relate to ‘behaviour’ or ‘outcomes’, check the outcomes reported in the Results section; if still unclear, code both (see 1.1). When set goals/targets (outcome) are presented alongside feedback, consider coding 1.6 Discrepancy between current behaviour and goal.  Heuristics informed by Presseau et al. (2015) | *"Targets were set at 7% for HbA1c, 130 mmHg for SBP and 100 mg/dl for LDL-C"* (Goderis, 2010) |
| **1.4 Action planning** | Prompt detailed planning of performance of the behaviour (must include at least one of context, frequency, duration and intensity). Context may be environmental (physical or social) or internal (physical, emotional or cognitive) (includes ‘Implementation Intentions’).  Note: evidence of action planning does not necessarily imply goal setting, only code latter if sufficient evidence. | Action planning is coded when HCPs are prompted or instructed to develop a plan to initiate a new clinical activity or process in order to achieve the desired behaviour and/or outcome. As per the original taxonomy, it must specify context, frequency, duration, and/or intensity. Code **1.2 Problem solving** for coping planning - planning how to support an existing clinical activity or process. Code **4.1 Instruction on how to perform the behaviour** if the feedback recipient was not involved in developing the action plan.  ***Key words***: plans; descriptions of plans in terms of who-when-where-how or if-then | *"Immediately after, a brief meeting took place with lead consultants and other key individuals to formulate a local action plan"* (Foy, 2004)  *"... person observed was helped formulate an action plan to improve behaviour. For example, when a healthcare worker didn’t clean hands after touching patient equipment but not the patient, the action was set as ‘‘X will use alcohol hand-rub even if only touching patient equipment’’."* (Fuller, 2012) |
| **1.5 Review behaviour goal(s)** | Review behaviour goal(s) jointly with the person and consider modifying goal(s) or behaviour change strategy in light of achievement. This may lead to re-setting the same goal, a small change in that goal or setting a new goal instead of (or in addition to) the first, or no change.   ***Note:*** *if goal specified in terms of behaviour, code 1.5; If goal unspecified, code 1.7; If discrepancy created consider also 1.6.* | The behaviour goal must be altered, re-set or agreed not to be changed to be coded. This can involve the refining or tailoring of goals to HCPs, which can be done in collaboration with HCPs or on their behalf.  Heuristics informed by Presseau et al. (2015) | *"... discussed the extent to which the plans were carried out, the impact of the change activities on the clinical decision making, and which aspects of clinical decision making needed further attention"* (Frijling, 2002)  *"… each practice reviewed their performance and set and reviewed goals speciﬁc to their individual circumstances and resources"* (Harris, 2015) |
| **1.6 Discrepancy between current behaviour and goal** | Draw attention to discrepancies between a person’s current behaviour (in terms of the form, frequency, duration, or intensity of that behaviour) and the person’s previously set outcome goals, behavioural goals or action plans (goes beyond self-monitoring of behaviour).   ***Note:*** *if discomfort is created only code 13.3 and not 1.6; If goals are modified, also code 1.5 and/or 1.7; If feedback is provided, also code 2.2.* | Code when HCP behaviour is contrasted with a 'standard' (e.g., guideline recommendations) in order to bring about change. Can be coded in instances where goal setting with HCPs is not explicit. Code **6.2 Social Comparison** when comparison is made with peers as opposed to a 'standard'.  ***Key words***: benchmark reports; guidelines IF current practice was fed back and compared against standards outlined in guidelines | *“... provided information on compliance with ﬁve appropriate-use criteria”* (Blais, 2008)   *"Each view displays a clinician’s performance against his or her clinic peers and* ***against national benchmarks****."* (Linder, 2010)  *"Reports included comparison against target benchmarks"* (Wiggers, 2017) |
| **1.9 Commitment** | Ask the person to affirm or reaffirm statements indicating commitment to change the behaviour.  ***Note:*** *if defined in terms of the behaviour to be achieved also code 1.1, Goal setting (behaviour).* | Code when the HCP is asked to affirm or reaffirm statements indicating commitment to change the behaviour, either in writing or verbally. For example, if HCPs are asked to sign an action plan.  ***Key words***: signing action plans | *"sign a commitment to change"* (Lafata, 2007)  *"The public commitment asked each of the participating village doctors in the intervention group to sign a letter of commitment and made the signed letter public by posting it on the walls of his or her clinic and printing it on the back of the patient takeaway information leaflet."* (Shen, 2018) |
| **2.0 Feedback (unspecified)** | Monitor and provide feedback.  Note: if Biofeedback, code only 2.6 and not 2.2; If feedback is on behaviour, code only 2.2; If feedback is on outcome(s) of behaviour, code only 2.7; If there is no clear evidence that feedback was given, code 2.1; If feedback on behaviour is evaluative e.g. praise, also code 10.4. | Feedback (unspecified) was not in the original taxonomy. It was added because due to poor reporting, it was not always clear from the Method or Results if the feedback related to outcome or behaviour, for example, some studies simply reported that they conducted an audit and feedback intervention.  ***Key words***: audit and feedback | *"Audit and feedback"* (Bahrami, 2004)  *"Each practice also received quarterly audit-and-feedback progress reports"* (Yano, 2008) |
| **2.1 Monitoring of behaviour by others without feedback** | Observe or record behaviour with the person’s knowledge as part of a behaviour change strategy.  ***Note:*** *if monitoring is part of a data collection procedure rather than a strategy aimed at changing behaviour, do not code. If feedback given, code only 2.2 and not 2.1; If monitoring outcome(s) code 2.5; If self-monitoring behaviour, code 2.3.* | Code if HCPs are informed that their behaviour is being monitored (e.g., prior to randomisation), but then they are not provided with feedback. Do not code routine data collection or if data is being collected for outcome measurement only. Will most likely be coded if described in the 'intervention' section as opposed to the 'outcome measurement' section.   Use supporting content to determine whether it is outcome and/or behaviour being monitored. | *"Hand-hygiene observation of a ward area for 20 minutes, recording the hand-hygiene behaviour of all healthcare workers entering that area (group compliance). Poor practice was documented but feedback was not given at the time."* (Fuller, 2012) |
| **2.2 Feedback on behaviour** | Monitor and provide informative or evaluative feedback on performance of the behaviour (e.g. form, frequency, duration, intensity).  ***Note:*** *if Biofeedback, code only 2.6 and not 2.2; If feedback is on outcome(s) of behaviour, code 2.7; If there is no clear evidence that feedback was given, code 2.1; If feedback on behaviour is evaluative e.g. praise, also code 10.4.* | Involves providing feedback on HCP behaviours such as screening rates and prescribing patterns. This differs to feedback on outcomes, which commonly relates to clinical outcomes such as presence or absence of a clinician condition. This code implies a monitoring process providing the feedback data.  ***Key words***: screening rates, prescription patterns | *"... involved an audit on indicators of pneumonia care with feedback delivered every month, using a specific pneumonia feedback sheet provided in both graphical and text-based formats"* (Ayieko, 2019)  *"Sites were provided with aggregated site-speciﬁc data on their use of antibiotics for acute respiratory tract infections in the pre-intervention winter year"* (Metlay, 2007) |
| **2.3 Self-monitoring of behaviour** | Establish a method for the person to monitor and record their behaviour(s) as part of a behaviour change strategy.  Note: if monitoring is part of a data collection procedure rather than a strategy aimed at changing behaviour, do not code; If monitoring of outcome of behaviour, code 2.4; If monitoring is by someone else (without feedback), code 2.1. | Self-monitoring of HCP behaviour requires the creation or active retrieval of information about the HCP's own behaviour. It may involve completing a checklist after a consultation has taken place; or logging into a dashboard to retrieve up-to-date information about their clinical behaviour, beyond the bare minimum (i.e., more frequently than what is required as part of the feedback process).  ***Key words***: auditing or completion of records about own behaviour; checklists IF used after consultation | *"... an active monitoring sheet designed to improve identification and documentation of signs and symptoms associated with the diagnosis of UTIs"* (Nace, 2020)  *"Daily each item is to be checked, initialed and dated upon completion by the staff on a daily basis"* (Wang, 2018) |
| **2.4 Self-monitoring of outcome(s) of behaviour** | Establish a method for the person to monitor and record the outcome(s) of their behaviour as part of a behaviour change strategy.  ***Note:*** *if monitoring is part of a data collection procedure rather than a strategy aimed at changing behaviour, do not code; If monitoring behaviour, code 2.3; If monitoring is by someone else (without feedback), code 2.5.* | Self-monitoring of outcomes of behaviour requires the creation or active retrieval of information about clinical or financial outcomes related to the HCP's own behaviour. It may involve completing a checklist after a consultation has taken place; or logging into a dashboard to retrieve up-to-date information about outcomes, beyond the bare minimum (i.e., more frequently than what is required as part of the feedback process).  ***Key words***: auditing or completion of records about clinical or financial outcomes of own behaviour; checklists IF used after consultation | *"2.4 Self-monitoring of outcome(s) of behaviour, e.g. presentation of compliance rates and discussion of options for monitoring on wards"* (von Lengerke et al., 2019) |
| **2.7 Feedback on outcome(s) of behaviour** | Monitor and provide feedback on the outcome of performance of the behaviour.  ***Note:*** *if Biofeedback, code only 2.6 and not 2.7; If feedback is on behaviour code 2.2; If there is no clear evidence that feedback was given code 2.5; If feedback on behaviour is evaluative e.g. praise, also code 10.4.* | Involves providing feedback on outcomes of HCP behaviours. The outcome is often a clinical outcome, at either the HCP or patient level, but could also be a financial outcome. This differs to feedback on behaviour, which commonly relates to screening rates or prescribing rates. This code implies a monitoring process providing the feedback data.  Heuristics informed by Presseau et al. (2015) | *"Key measures of guideline compliance such as time to AT, time to source control, and compliance with appropriate taking of blood cultures, along with patient outcomes (including mortality, ICU and hospital length of stay) were summarized into individual reports"* (Bloos, 2017)  *"The average drug cost for the participant and the peer group defined above was also shown"* (Hux, 1999) |
| **3.1 Social support (unspecified)** | Advise on, arrange, or provide social support (e.g. from friends, relatives, colleagues, ‘buddies’ or staff) or non-contingent praise or reward for performance of the behaviour. It includes encouragement and counselling, but only when it is directed at the behaviour. | Code when there is no explicit mention of the type of support provided, for example, if coaching or supervision is offered and there is no detail about the nature of the support provided. The support can be provided from a colleague or a member of the research team. Don't confuse with delivery (e.g. one off education sessions delivered by HCPs). Consider whether the social support is delivered over time (e.g. having somebody on-site working with HCPs to achieve target behaviour).  ***Key words***: coaching, supervision | *"Afterwards, the GPs were contacted by telephone by a senior clinical pharmacologist (JS) to discuss any uncertainties concerning the recommendations given"* (Bregnhøj, 2009)  *"... clinicians could collaboratively consult ADHD experts via a health system online networking site or private email/telephone conversation"* (Fiks, 2017) |
| **3.2 Social support (practical)** | Advise on, arrange, or provide practical help (e.g. from friends, relatives, colleagues, ‘buddies’ or staff) for performance of the behaviour.  Note: if emotional, code 3.3; If general or unspecified, code 3.1; If only restructuring the physical environment or adding objects to the environment, code 12.1; Adding objects to the environment; attending a group or class and/or mention of ‘follow-up’ does not necessarily apply this BCT, support must be explicitly mentioned. | Code when a team member or researcher facilitates/helps an existing HCP achieve the target behaviour or outcome. Also code 12.1 Restructuring the Social Environment if a new person is introduced or an existing team member changes roles, in order to provide this support. Only code 12.1 if the purpose of the staff restructure was not to provide support. Don't confuse with delivery (e.g. one off education sessions delivered by HCPs). Consider whether the social support is delivered over time (e.g. having somebody on-site working with HCPs to achieve target behaviour).  Heuristics informed by Presseau et al. (2015) | *"Stock cards were filled by facilitators on a daily basis and they were trained to recognize the minimum stock levels to prevent supply stock out"* (Althabe, 2019)  *"Selected staff from each intervention home were designated as infection control link workers, their role being to reinforce all aspects of good infection control throughout the study"* (Baldwin, 2010) |
| **3.3 Social support (emotional)** | Advise on, arrange, or provide emotional social support (e.g. from friends, relatives, colleagues, ‘buddies’ or staff) for performance of the behaviour.  Note: if practical, code 3.2, Social support (practical); if unspecified, code 3.1, Social support (unspecified) | Code when a team member or researcher provides emotional support to the HCP as they carry out the target behaviour, commonly through the use of empathetic communication. | *"3.3 Social support (emotional), e.g. active listening in feedback discussions to evoke reflection on balancing benefits and costs"* (von Lengerke et al., 2019) |
| **4.0 Education (unspecified)** | Provision of education and/or training, without explicit description of content. | Education (unspecified) was not in the original taxonomy. It was added because due to poor reporting, it was not always clear if the education or training incorporated instruction on how to perform the behaviour. Frequently, authors referred to the provision of educational materials, learning modules, evidence summaries, and academic detailing, without any additional information. Try not to conflate with 4.1.  Often see BCTs like 4.1 embedded within an educational training/program/module (which is poorly reported). In such cases, consider coding 4.0 in addition to other BCTs (e.g. 4.1).  ***Key words***: training, educational materials, learning modules, academic detailing, evidence summaries, frequently asked questions | *"summaries of supporting medical literature"* (Bonds, 2009)  *"At 6 months, clinics received a 30-min “booster” training."* (Mertens, 2015)  *"contained brief, evidence-based clinical messages"* (Willis, 2020) |
| **4.1 Instruction on how to perform the behaviour** | Advise or agree on how to perform the behaviour (includes ‘Skills training’).  Note: when the person attends classes such as exercise or cookery, code 4.1, 8.1 and 6.1. | Instruction for HCPs typically comes in the form of guidelines, decision aids, job aids, and pocket cards. Also code 5.1 Information about health consequences if these forms of instruction explain the health consequences associated with not following the instructions. Also code 9.1 Credible source if the instructions are endorsed by a professional or academic body that is perceived as credible. Also code 7.1 Prompts/cues if the instructions are positioned to trigger a target behaviour at the time the behaviour is to occur.  Try not to conflate with 4.0.  ***Key words***: guidelines, recommendations, decision aids, job aids, pocket cards, skills training, newsletters IF specifically referred to tips, reference to guidelines, etc | *"Reminders, created by the selected facilitators and the rest of ANC health providers, consisted on simple messages and figures to remind prenatal health providers on how to conduct syphilis screening procedures and how to provide treatment for those women found positive at the first ANC visit"* (Althabe, 2019)  *"The practical goals and guidelines section summarized current recommendations by the American Diabetes Association^19^ and the Joint National Committee on Prevention, Detection, Evaluation, and Treatment of High Blood Pressure"* (Estrada, 2011) |
| **4.2 Information about antecedents** | Provide information about antecedents (e.g. social and environmental situations and events, emotions, cognitions) that reliably predict performance of the behaviour. | As per original taxonomy | *“Module 3 presented information on the possible impact of medical malpractice concerns on recall rates.”* (Carney et al., 2012) |
| **4.4 Behavioural experiments** | Advise on how to identify and test hypotheses about the behaviour, its causes and consequences, by collecting and interpreting data. | As per original taxonomy | *“4.4 Behavioural experiments, e.g. Fluorescence behaviour training by fluorescence methods using ultraviolet light boxes.”* (von Lengerke et al., 2019) |
| **5.1 Information about health consequences** | Provide information (e.g. written, verbal, visual) about health consequences of performing the behaviour.  Note: consequences can be for any target, not just the recipient(s) of the intervention; emphasizing importance of consequences is not sufficient; If information about emotional consequences, code 5.6; If about social and environmental consequences code 5.3; If unspecified, code 5.1. | Code when the HCP is informed of the anticipated health outcomes for the patient associated with target clinical processes (e.g. infections, transmission, and consequences of antibiotics/AMR). Commonly, this may be presented as an increase or decrease of health risk associated with a particular clinical behaviour; or information about a specific topic or condition. | *"The modules emphasized that (1) young, sexually active women are at high risk for asymptomatic infection that may lead to future serious health consequences ..."* (Allison, 2005)  *"The meeting included background information on the causes and consequences of polypharmacy ..."* (Bregnhøj, 2009) |
| **5.2 Salience of consequences** | Use methods specifically designed to emphasise the consequences of performing the behaviour with the aim of making them more memorable (goes beyond informing about consequences).  Note: if information about consequences, also code 5.1, Information about health consequences, 5.6, Information about emotional consequences or 5.3, Information about social and environmental consequences | Code when information about the consequences of performing a behaviour is presented to the HCP using methods designed to generate an emotive response. It must go beyond information exchange. | *“public health catastrophe”* (Hallsworth et al., 2016) |
| **5.3 Information about social and environmental consequences** | Provide information (e.g. written, verbal, visual) about social and environmental consequences of performing the behaviour.  Note: consequences can be for any target, not just the recipient(s) of the intervention; If information about health or unspecified consequences, code 5.1; If about emotional consequences, code 5.6. | Code when the HCP is informed of the anticipated social and environmental outcomes associated with target clinical processes, for example, cost savings, patient safety, equitable access. | *"… each physician would be fiscally responsible for their prescribing"* (Mainous, 2000)  *"… antibiotic-related problems (resistance, medicalization, unnecessary side effects and costs) were discussed"* (van der Velden, 2016) |
| **5.6 Information about emotional consequences** | Provide information (e.g. written, verbal, visual) about emotional consequences of performing the behaviour.  Note: consequences can be related to emotional health disorders (e.g. depression, anxiety) and/or states of mind (e.g. low mood, stress); not including 5.5, Anticipated regret; consequences can be for any target, not just the recipient(s) of the intervention; if information about health consequences code 5.1, Information about health consequences; if about social, environmental or unspecified code 5.3, Information about social and environmental consequences. | Code when the HCP is informed of the anticipated emotional outcomes associated with target clinical processes, for example, reductions in staff stress or improved mood. | *“5.6 Information about emotional consequences, e.g. knowledge transfer on psychological consequences of Nis”* (von Lengerke et al., 2019) |
| **6.1 Demonstration of the behaviour** | Provide an observable sample of the performance of the behaviour, directly in person or indirectly e.g. via film, pictures, for the person to aspire to or imitate (includes ‘Modelling’).  Note: if advised to practice, also code, 8.1; If provided with instructions on how to perform, also code 4.1. | Code when there is explicit mention of ‘demonstration’ or ‘modelling’. Reference to instruction, guidance, or leaflets is not sufficient and should be coded as **4.1 Instruction to perform the behaviour**.  ***Key words***: practical demonstration, modelling  Heuristics informed by Davey et al. (2017) | *“Practical demonstrations on hand hygiene and decontamination of equipment and the environment were also provided during the session.”* (Baldwin, 2010)  *"... the goal of the local champion component was to provide role-modeling and promote attitudinal change in the ICU"* (Curtis, 2011) |
| **6.2 Social comparison** | Draw attention to others’ performance to allow comparison with the person’s own performance.  Note: being in a group setting does not necessarily mean that social comparison is actually taking place. | Code when HCP behaviour is contrasted with that of identified or deidentified peers, including other HCPs, hospitals, or clinics, in order to bring about change. Code **1.6 Discrepancy between current behaviour and goal** when comparison is made with a 'standard' as opposed to peers.  ***Key words***: feedback graphs comparing GP behaviour with that of other GP clinics, outlier tables  Heuristics informed by Davey et al. (2017) | *"prescribers received an email that showed them the amount of prescribing by center, and identified the top prescriber in the state mental health system"* (Brunette, 2015)  *"The information was shown graphically; with a display of all intervention practices (indicated by code) for comparison"* (Halterman, 2014) |
| **6.3 Information about others’ approval** | Provide information about what other people think about the behavior. The information clarifies whether others will like, approve or disapprove of what the person is doing or will do. | Code when HCPs are informed that their managers, peers, and/or patients are in favour of the change to clinical processes. | *“6.3 Information about others’ approval, e.g. reflection of perceived recognition by superiors for compliance as assessed in survey”* (von Lengerke et al., 2019) |
| **7.1 Prompts/cues** | Introduce or define environmental or social stimulus with the purpose of prompting or cueing the behaviour. The prompt or cue would normally occur at the time or place of performance.  Note: when a stimulus is linked to a specific action in an if-then plan including one or more of frequency, duration or intensity also code 1.4. | Code if physical objects or electronic systems (e.g. signs, pocket-books, checklists) are added or modified to trigger performance of a targeted behaviour at the time the behaviour is to occur (e.g., in consultation room, ward, chart). Do not code as 12.5 Adding objects to the environment as this refers to mode of delivery and not a unique BCT.  ***Key words***: reminders, checklists IF used before or during consultation | *"Identified patients were flagged by the tool as needing review"* (Dreischulte, 2016)  *"a computer alert prompting testing of eligible patients"* (Hocking, 2018)  *"A chart reminder system was designed to prompt physicians to consider prescribing therapy on a timely basis."* (Leviton, 1999) |
| **8.1 Behavioural practice/rehearsal** | Prompt practice or rehearsal of the performance of the behaviour one or more times in a context or at a time when the performance may not be necessary, in order to increase habit and skill.  Note: if aiming to associate performance with the context, also code 8.3. | As per original taxonomy | *"… hands-on experience with an insulin pen"* (Harris, 2013)  *"exercises on hand hygiene in line with guidelines regarding glove use"* (von Lengerke, 2019) |
| **8.2 Behaviour substitution** | Prompt substitution of the unwanted behaviour with a wanted or neutral behaviour.  Note: if this occurs regularly, also code 8.4, Habit reversal. | As per original taxonomy | *“The main focus of the guidelines was to restrict prescriptions to bacterial infections and to preferentially prescribe narrow-spectrum antibiotics, namely penicillins for RTIs and trimethoprim/sulfamethoxazole for uncomplicated lower UTIs.”* (Hürlimann et al., 2015) |
| **8.6 Generalisation of target behaviour** | Advise to perform the wanted behaviour, which is already performed in a particular situation, in another situation. | As per original taxonomy | *“8.6 Generalisation of target behaviour, e.g. transfer of problem-solving approaches across indications”* (von Lengerke et al., 2019) |
| **8.7 Graded tasks** | Set easy-to-perform tasks, making them increasingly difficult, but achievable, until behaviour is performed. | As per original taxonomy | *“8.7 Graded tasks, e.g. focusing on individual indications such as before aseptic procedures”* (von Lengerke et al., 2019) |
| **9.1 Credible source** | Present verbal or visual communication from a credible source in favour of or against the behaviour.  Note: code this BCT if source generally agreed on as credible e.g., health professionals, celebrities or words used to indicate expertise or leader in field and if the communication has the aim of persuading; If information about health consequences, also code 5.1; If about emotional consequences, also code 5.6; If about social, environmental or unspecified consequences also code 5.3. | Code if guidelines, education materials, or feedback reports are endorsed by respected peers or professional/academic bodies that are perceived as credible (e.g., National Diabetes Society). This would frequently be coded if experts in the field or logos of professional/academic bodies are included on materials or feedback reports.  ***Key words***: name of professional association / academic body, expert | *"... provided by certified instructors from the Society of Obstetricians and Gynaecologists of Canada"* (Chaillet, 2015)  *“opinion leader–led feedback to providers”* (Lakshminarayan, 2010)  *"The CEO of The Permanente Medical Group (which employs the organization’s physicians) endorsed the project"* (Mertens, 2015) |
| **9.2 Pros and cons** | Advise the person to identify and compare reasons for wanting (pros) and not wanting to (cons) change the behaviour (includes ‘Decisional balance’).  Note: if providing information about health consequences, also code 5.1; If providing information about emotional consequences, also code 5.6; If providing information about social, environmental or unspecified consequences also code 5.3. | As per original taxonomy | *"9.2 Pros and cons, e.g. discussing effects of compliance and noncompliance"* (von Lengerke, 2019) |
| **10.1 Material incentive (behaviour)** | Inform that money, vouchers or other valued objects will be delivered if and only if there has been effort and/or progress in performing the behaviour (includes ‘Positive reinforcement’).  Note: if incentive is social, code 10.5; If unspecified code 10.6 and not 10.1; If incentive is for outcome, code 10.8; If reward is delivered also code one of: 10.2, 10.3, 10.4, 10.9, or 10.10. | Code if HCPs are informed prior to the intervention that valued objects (e.g. funding or accredited points linked to performance indicators) will be provided, contingent on attempts to conduct the target behaviour. For example, if HCPs are informed that they will receive payment for achieving screening rate targets. If it is not clear when HCPs were notified of the material reward, code **10.2 Material reward (behaviour).** Do not code incentives/rewards designed to promote research participation.  ***Key words***: accredited points (professional association) IF notified prior to the intervention  Heuristics informed by Davey et al. (2017) | *"Intervention group participants received up to five incentive payments in their paychecks approximately every four months and were notified each time a payment was posted."* (Petersen, 2013) |
| **10.2 Material reward (behaviour)** | Arrange for the delivery of money, vouchers or other valued objects if and only if there has been effort and/or progress in performing the behaviour (includes ‘Positive reinforcement’).  ***Note:*** *If reward is social, code 10.4, Social reward, if unspecified code 10.3, Nonspecific reward, and not 10.1, Material reward (behaviour); if reward is for outcome, code 10.10, Reward (outcome). If informed of reward in advance of rewarded behaviour, also code one of: 10.1, Material incentive (behaviour); 10.5, Social incentive; 10.6, Non-specific incentive; 10.7, Self-incentive; 10.8, Incentive (outcome).* | Code if HCPs are provided with valued objects (e.g. funding or accredited points linked to performance indicators), contingent on attempts to conduct the target behaviour. For example, if HCPs receive payment for achieving screening rate targets. Do not code incentives/rewards designed to promote research participation. In instances where they report delivery of the reward, typically in results section (e.g. # of HCPs who achieved reward, amount paid out in rewards), do not capture, no need to double code.  ***Key words***: accredited points (professional association) IF NOT notified prior to receipt of points, certificate of participation/completion | *"... practices received financial incentives in the form of an initial fixed payment of £350 ($600 U.S.) and a payment of £15 ($25 U.S.) for every patient for whom the targeted high-risk prescribing was reviewed during the intervention period"* (Dreischulte, 2016)  *"Physicians assigned to the bonus and feedback group were eligible to receive financial bonuses based on patients' up-todate coverage for DTP and Haemophilus influenzae type b (Hib), OPV, and MMR."* (Fairbrother, 1999) |
| **10.4 Social reward** | Arrange verbal or non-verbal reward if and only if there has been effort and/or progress in performing the behaviour (includes ‘Positive reinforcement’).  ***Note:*** *if reward is material, code 10.2; If unspecified code 10.3 and not 10.4; If reward is for outcome code 10.10; If informed of reward in advance of rewarded behaviour also code one of: 10.1, 10.5, 10.6, 10.7 or 10.8.* | A social reward for HCPs commonly occurs in the form of positive reinforcement in a feedback report, for example, 'well done!' or a green smiley face.  ***Key words***: "Well done!", green smiley faces | *"If compliance was 100%, the staff member was praised"* (Fuller, 2012)  *"newsletters highlighted practice successes"* (Yano, 2008) |
| **10.5 Social incentive** | Inform that a verbal or non-verbal reward will be delivered if and only if there has been effort and/or progress in performing the behaviour (includes ‘Positive reinforcement’)  Note: if incentive is material, code 10.1, Material incentive (behaviour), if unspecified code 10.6, Non-specific incentive, and not 10.5, Social incentive; if incentive is for outcome code 10.8, Incentive (outcome). If reward is delivered also code one of: 10.2, Material reward (behaviour); 10.3, Non-specific reward; 10.4, Social reward, 10.9, Self-reward; 10.10, Reward (outcome). | A social incentive for HCPs commonly occurs in the form of *anticipated* positive reinforcement, for example, knowing they or their team would be recognised in a professional context (newsletter, team meeting, feedback report) if they were to carry out the clinical process/behaviour. | *“Incentives to conduct SBIRT were limited to clinic recognition in the quality feedback reports (see “quality feedback reports” above) for high performing clinics.”* (Mertens et al., 2015) |
| **10.8 Incentive (outcome)** | Inform that a reward will be delivered if and only if there has been effort and/or progress in achieving the behavioural outcome (includes ‘Positive reinforcement’)  Note: this includes social, material, self- and non-specific incentives for outcome; if incentive is for the behaviour code 10.5, Social incentive, 10.1, Material incentive (behaviour), 10.6, Non-specific incentive or 10.7, Self-incentive and not 10.8, Incentive (outcome). If reward is delivered also code one of: 10.2, Material reward (behaviour); 10.3, Non-specific reward; 10.4, Social reward, 10.9, Self-reward; 10.10, Reward (outcome). | An incentive is offered to HCPs if they were to achieve or make progress towards achieving their behavioural outcomes. The incentives would typically be social (e.g., professional recognition in team meeting / newsletter) or material (e.g., financial reward) in nature. | *“primary care providers were each eligible for $75 three and six months after enrollment in the program if the patient’s hemoglobin A1c went down by at least 0.5 points from baseline or achieved a value of 9.0 or lower”* (Navathe et al., 2020) |
| **12.1 Restructuring the physical environment** | Change, or advise to change the physical environment in order to facilitate performance of the wanted behaviour or create barriers to the unwanted behaviour (other than prompts/cues, rewards and punishments).  ***Note:*** *this may also involve 12.3; If restructuring of the social environment code 12.2; If only adding objects to the environment, code 12.5.* | Code only if it is a change to an existing physical structure, for example, a ward restructure. If something physically NEW is added to the environment that facilitates the behaviour change, code as **12.5 Adding objects to the environment**. Changes to electronic systems are changes to mode of delivery and should not be coded as a BCT.  ***Key words***: ward restructure, reposition objects  Heuristics informed by Presseau et al. (2015) | *"making structural changes to the clinic to improve blood pressure control"* (Bonds, 2009)  *"The CKD registry was designed to alert practice teams of a patient’s CKD-relevant information"* (Tuot, 2018) |
| **12.2 Restructuring the social environment** | Change, or advise to change the social environment in order to facilitate performance of the wanted behaviour or create barriers to the unwanted behaviour (other than prompts/cues, rewards and punishments).  ***Note:*** *this may also involve 12.3; If also restructuring of the physical environment also code 12.1.* | Code when the social environment changes to help care being provided. Code when someone new takes on responsibility for providing care that was previously provided by someone else, or someone part of the existing HCP team takes on new care responsibilities. Can be someone added to a core team or shifting care to someone outside the core team (e.g. pharmacist). It may involve the selection or identification of opinion leaders, local facilitators, or champions; also code **9.1 Credible source** for local champions, and possibly local facilitators and opinion leaders, if they are well respected among target HCPs. Also code **Social support (3.1 or 3.2 or 3.3)** if the restructure was intended to provide support.  ***Key words***: local facilitator, opinion leader, local champion  Heuristics informed by Presseau et al. (2015) | *"Selected staff from each intervention home were designated as infection control link workers, their role being to reinforce all aspects of good infection control throughout the study."* (Baldwin, 2010)  *"The second component is training local leaders, including a primary care clinician, a nursing supervisor, and a mental health specialist, to implement the interventions."* (Wells, 2000) |
| **12.5 Adding objects to the environment** | Add objects to the environment in order to facilitate performance of the behaviour.  ***Note:*** *Provision of information (e.g. written, verbal, visual) in a booklet or leaflet is insufficient. If this is accompanied by social support, also code 3.2; If the environment is changed beyond the addition of objects, also code 12.1.* | Code for the addition of new physical or virtual (software) aimed at facilitating behaviour change. If something existing is changed, code **12.1 Restructuring the physical environment.** Posters, flyers, electronic cards, or checklists should be coded as **7.1 prompts/cues** if added to trigger performance of a targeted behaviour at the time the behaviour is to occur; otherwise code 12.5. Decision support systems/algorithms should only be coded as **4.1 instruction for performing the behaviour.**  Heuristics informed by Presseau et al. (2015) | *"provided with automatic blood pressure machines"* (Bonds, 2009)  *"“Prescription” pads providing written recommendations for symptomatic treatment of viral infections were adapted from previous CDC-sponsored campaigns"* (Finkelstein, 2008) |
| **13.1 Identification of self as role model** | Inform that one's own behaviour may be an example to others. | As per original taxonomy | *“13.1 Identification of self as role model, e.g. illustration and discussion of the function of role models in hand hygiene compliance”* (von Lengerke et al., 2019) |
| **13.2 Framing/reframing** | Suggest the deliberate adoption of a perspective or new perspective on behaviour (e.g. its purpose) in order to change cognitions or emotions about performing the behaviour (includes ‘Cognitive structuring’).  Note: If information about consequences, then code 5.1, Information about health consequences, 5.6, Information about emotional consequences or 5.3, Information about social and environmental consequences instead of 13.2, Framing/reframing. | As per original taxonomy | *“13.2 Framing/Reframing, e.g. raising the issue of compliance as a team task (team cooperation)”* (von Lengerke et al., 2019) |
| **14.6 Situation-specific reward** | Arrange for reward following the behaviour in one situation but not in another (includes ‘Discrimination training’).  Note: also code one of 10.2, Material reward (behaviour); 10.3, Non-specific reward; 10.4, Social reward, 10.9, Self-reward; 10.10, Reward (outcome). | As per original taxonomy | *“14.6 Situation-specific reward, e.g. certification of ward with highest compliance with the trial”* (von Lengerke et al., 2019) |
| **15.1 Verbal persuasion about**  **capability** | Tell the person that they can successfully perform the wanted behaviour, arguing against self-doubts and asserting that they can and will succeed. | As per original taxonomy | *“15.1 Verbal persuasion about capability, e.g. discussion of positive compliance development”* (von Lengerke et al., 2019) |
| **15.3 Focus on past success** | Advise to think about or list previous successes in performing the behaviour (or parts of it). | As per original taxonomy | *“15.3 Focus on past success, e.g. discussion of best year”* (von Lengerke et al., 2019) |
