# Supplementary material for: Identifying behaviour change techniques in 287 randomized controlled trials of audit and feedback interventions targeting practice change among healthcare professionals
Source: Implement Sci. 2023 Nov 21;18:63. doi: 10.1186/s13012-023-01318-8 (PMC10664600; doi:10.1186/s13012-023-01318-8)
Supplement: Supplementary file 3 — Additional file 3: Appendix 3. Descriptive statistics for BCT frequencies by study year of publication. [file 13012_2023_1318_MOESM3_ESM.docx]

***Appendix 3.* Descriptive statistics for BCT frequencies by study year of publication.**

Frequency distribution graphs to remove outliers for Treatment arms.

Frequency distribution graphs to remove outliers in Control/comparator arms.

Descriptive stats for Treatment arms (after removing outliers; n=3)

| Year | Number of studies published | Min BCTs | Max BCTs | Mean | SD |
| --- | --- | --- | --- | --- | --- |
| 1980 | 1 | 3 | 4 | 4 | 0.7 |
| 1982 | 2 | 1 | 5 | 3 | 2.8 |
| 1983 | 1 | 3 | 3 | 3 | N/A |
| 1984 | 1 | 2 | 2 | 2 | N/A |
| 1985 | 2 | 3 | 5 | 4 | 1.2 |
| 1986 | 2 | 3 | 4 | 3 | 0.6 |
| 1987 | 1 | 2 | 3 | 3 | 0.7 |
| 1988 | 2 | 4 | 7 | 5 | 1.7 |
| 1990 | 3 | 1 | 5 | 3 | 1.3 |
| 1991 | 2 | 2 | 5 | 3 | 1.4 |
| 1992 | 1 | 3 | 3 | 3 | N/A |
| 1993 | 1 | 4 | 4 | 4 | 0.0 |
| 1994 | 2 | 5 | 6 | 5 | 0.5 |
| 1995 | 4 | 3 | 9 | 6 | 2.7 |
| 1996 | 1 | 4 | 4 | 4 | 0.0 |
| 1997 | 3 | 3 | 7 | 5 | 2.1 |
| 1998 | 8 | 2 | 9 | 6 | 2.6 |
| 1999 | 11 | 3 | 6 | 4 | 0.8 |
| 2000 | 5 | 3 | 6 | 5 | 1.3 |
| 2001 | 12 | 3 | 10 | 6 | 2.2 |
| 2002 | 5 | 1 | 9 | 4 | 2.9 |
| 2003 | 10 | 1 | 7 | 4 | 1.9 |
| 2004 | 13 | 1 | 7 | 4 | 1.7 |
| 2005 | 7 | 2 | 7 | 4 | 1.7 |
| 2006 | 8 | 1 | 9 | 5 | 2.3 |
| 2007 | 10 | 3 | 8 | 5 | 1.4 |
| 2008 | 11 | 1 | 11 | 6 | 2.7 |
| 2009 | 9 | 1 | 9 | 4 | 1.9 |
| 2010 | 6 | 4 | 8 | 6 | 1.7 |
| 2011 | 11 | 2 | 11 | 6 | 3.0 |
| 2012 | 5 | 5 | 6 | 6 | 0.5 |
| 2013 | 11 | 2 | 13 | 6 | 2.8 |
| 2014 | 9 | 3 | 8 | 5 | 1.6 |
| 2015 | 16 | 2 | 12 | 8 | 3.4 |
| 2016 | 20 | 1 | 11 | 5 | 2.8 |
| 2017 | 8 | 4 | 11 | 6 | 2.1 |
| 2018 | 17 | 3 | 11 | 6 | 2.0 |
| 2019 | 16 | 2 | 13 | 6 | 3.0 |
| 2020 | 7 | 4 | 12 | 8 | 3.1 |

Descriptive stats for Control/comparator groups (after removing outliers; n=2

| Year | Number of studies published | Minimum BCTs | Max BCTs | Mean | SD |
| --- | --- | --- | --- | --- | --- |
| 1980 | 1 | 0 | 0 | 0 | N/A |
| 1982 | 2 | 0 | 0 | 0 | 0.0 |
| 1983 | 1 | 0 | 0 | 0 | N/A |
| 1984 | 1 | 0 | 0 | 0 | N/A |
| 1985 | 2 | 0 | 3 | 1 | 1.7 |
| 1986 | 2 | 0 | 2 | 1 | 1.2 |
| 1987 | 1 | 1 | 1 | 1 | N/A |
| 1988 | 2 | 0 | 3 | 2 | 1.7 |
| 1990 | 3 | 0 | 1 | 0 | 0.6 |
| 1991 | 2 | 0 | 2 | 1 | 1.4 |
| 1992 | 1 | 0 | 0 | 0 | N/A |
| 1993 | 1 | 0 | 0 | 0 | 0.0 |
| 1994 | 2 | 0 | 1 | 1 | 0.7 |
| 1995 | 4 | 0 | 2 | 0 | 0.8 |
| 1996 | 1 | 4 | 4 | 4 | N/A |
| 1997 | 3 | 1 | 4 | 2 | 1.5 |
| 1998 | 8 | 0 | 6 | 2 | 2.1 |
| 1999 | 11 | 0 | 3 | 1 | 1.1 |
| 2000 | 5 | 0 | 2 | 1 | 0.8 |
| 2001 | 12 | 0 | 8 | 2 | 2.9 |
| 2002 | 5 | 0 | 6 | 2 | 2.3 |
| 2003 | 10 | 0 | 2 | 0 | 0.6 |
| 2004 | 13 | 0 | 9 | 2 | 2.8 |
| 2005 | 7 | 0 | 4 | 1 | 1.5 |
| 2006 | 8 | 0 | 2 | 1 | 0.7 |
| 2007 | 10 | 0 | 5 | 1 | 1.7 |
| 2008 | 11 | 0 | 4 | 1 | 1.4 |
| 2009 | 9 | 0 | 4 | 1 | 1.7 |
| 2010 | 6 | 0 | 9 | 2 | 3.4 |
| 2011 | 11 | 0 | 6 | 2 | 2.2 |
| 2012 | 5 | 0 | 2 | 0 | 0.9 |
| 2013 | 11 | 0 | 9 | 3 | 2.8 |
| 2014 | 9 | 0 | 5 | 1 | 1.8 |
| 2015 | 16 | 0 | 6 | 2 | 2.4 |
| 2016 | 20 | 0 | 8 | 1 | 2.3 |
| 2017 | 8 | 0 | 1 | 0 | 0.3 |
| 2018 | 17 | 0 | 5 | 1 | 1.7 |
| 2019 | 16 | 0 | 5 | 1 | 1.5 |
| 2020 | 7 | 0 | 5 | 1 | 1.9 |
